# The Lévy flight foraging hypothesis: forgetting about memory may lead to false verification of Brownian motion

**DOI:** 10.1186/2051-3933-1-9

**Published:** 2013-10-14

**Authors:** Arild O Gautestad, Atle Mysterud

**Affiliations:** Centre for Ecological and Evolutionary Synthesis, Department of Biosciences, University of Oslo, P.O. Box 1066, Blindern, Oslo, NO-0316 Norway

**Keywords:** Optimal foraging, Lévy flight foraging hypothesis, Memory-influenced movement, Site fidelity, Statistical mechanics of movement, Scale-free space use

## Abstract

**Background:**

The Lévy flight foraging hypothesis predicts a transition from scale-free Lévy walk (LW) to scale-specific Brownian motion (BM) as an animal moves from resource-poor towards resource-rich environment. However, the LW-BM continuum implies a premise of memory-less search, which contradicts the cognitive capacity of vertebrates.

**Results:**

We describe methods to test if apparent support for LW-BM transitions may rather be a statistical artifact from movement under varying intensity of site fidelity. A higher frequency of returns to previously visited patches (stronger site fidelity) may erroneously be interpreted as a switch from LW towards BM. Simulations of scale-free, memory-enhanced space use illustrate how the ratio between return events and scale-free exploratory movement translates to varying strength of site fidelity. An expanded analysis of GPS data of 18 female red deer, *Cervus elaphus,* strengthens previous empirical support of memory-enhanced and scale-free space use in a northern forest ecosystem.

**Conclusion:**

A statistical mechanical model architecture that describes foraging under environment-dependent variation of site fidelity may allow for higher realism of optimal search models and movement ecology in general, in particular for vertebrates with high cognitive capacity.

**Electronic supplementary material:**

The online version of this article (doi:10.1186/2051-3933-1-9) contains supplementary material, which is available to authorized users.

## Background

**Figure 1:**
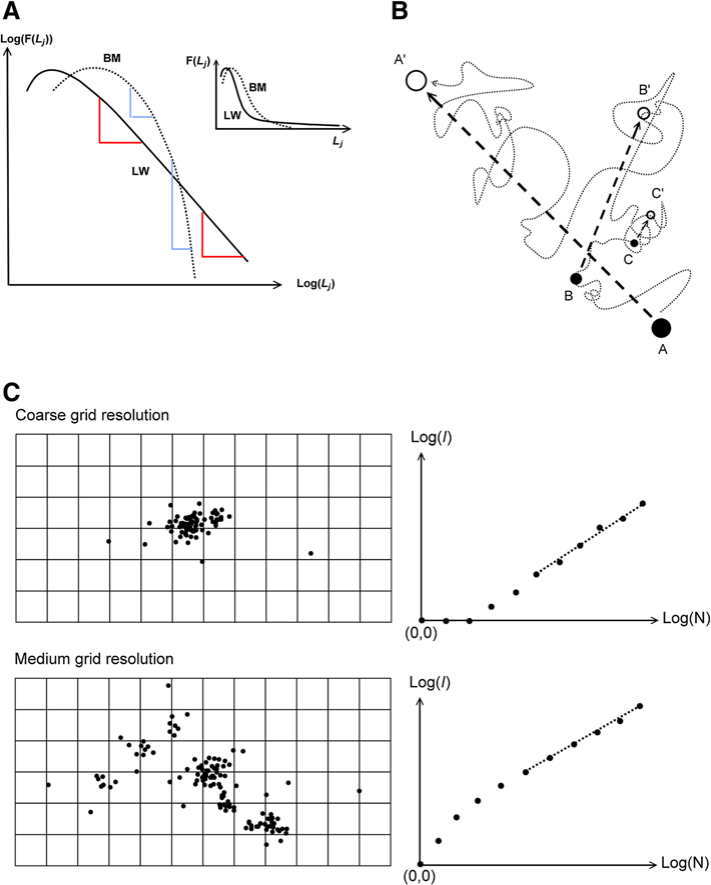
**The concepts of scaling and memory-influenced site fidelity. (A)** Lévy walk (LW) and Brownian motion (BM) may be characterized by the tail part of the distribution F(*L*_*j*_) of inter-fix steps lengths *L*, where *L*_*j*_ is a specific length range ( “bin size”). On a log-log scale *β* is expressing how “steeply” the frequency of larger steps fades, relative to any reference length *L*_*j*_, over the range of *j* where *β* is relatively constant. Since this ratio between two magnitudes of *L*_*j*_ is independent on which absolute size of *L*_*j*_ we choose for comparison (e.g., “meters” in both numerator and denominator cancels each other, leading to a dimensionless number), the movement is scale-free. For a scale-specific kind of movement like BM (dotted line), the distribution shows *β* increasing with increasing *j* with *β*>3. **(B)** Memory-influenced movement under the present model is conceptualized by three hypothetical goals along a spatial path. Long term goal (arrow towards target A’, to be reached within time t_a’_, decided at location A at time t_a_), medium term goal (B and B’) and short term goal (C and C’). The difference in time intervals for the three targets implies different process rates, and consequently an option to execute several goals at finer temporal scales for each goal at coarser scales. **(C)** The spatial scatters of two hypothetical sets of fixes illustrate a method to study scaling and site fidelity. Number of non-empty grid cells (*I*) will depend on both the grid resolution and the number of fixes (N) in the sample. The optimal resolution is found where the regression line may be interpolated close to (0,0); i.e., somewhere intermediate between the two examples. Linearity of the slope implies scale-free space use, and a magnitude intermediate between 1 and 0 (e.g., close to 0.5) indicates site fidelity and hence compliance with the non-Markovian framework.

Foraging theory aims to identify the complex mixture of behaviour and morphology most efficient to gather energy in different environments [1]. Identifying characteristics of efficient search is one important component in such an effort. The Lévy flight foraging (LFF) hypothesis extends the theory of optimal foraging by bringing a closer attention to the distinction between scale-free and scale-specific movement [2–4]. It has arisen as a result of a constructive interplay between simulation models identifying optimal space use tactics under given environmental conditions [3, 5] and empirical testing of these predictions on animal movement data [6–9]. In short, Lévy walk (LW, which may be considered synonymous with Lévy flight in the present context) exemplifies scale-free foraging (Figure 1A). In this case, the animal relates to its habitat in a complex manner – involving movement optimization over a range of scales. Many short movement bouts are stochastically interspersed by some long moves and occasionally some very long ones, owing to varying duration of directional persistence of successive displacements. The occasional very long steps of scale-free LW are identified as the most efficient in a resource-poor environment where food items or patches are randomly scattered and relatively unpredictable. Resource detection is then enhanced by a total movement path that is covering a large area for a given movement speed (path length per unit time).

LW contrasts with a more scale-specific and “fine-grained” kind of movement, Brownian motion (BM), which may be expected to arise in environments with more frequent responses to events and conditions within the perceptual range resulting in low degree of directional persistence of the path. One such event could for example be detection and handling of a food item, followed by a new displacement that is independent on the foregoing ones. The medium range steps are in this case rarer than for LW and the longest ones are completely absent. Consequently, the area covered during a given time period is small relative to LW under similar movement speed. A BM, which describes a stochastic process, will result from sampling an actual movement path at sufficiently large time intervals to ensure that successive relocations (fixes) provide inter-fix distances that reflect a sum of independent and intermediate movement events [5, 10, 11]. Whether the behavioural response to specific local situations (like the emergence of prey within the perceptual field) is determined by simple or complex rules, or whether the respective rules are characterized by a small or large degree of stochasticity, the direction from one fix to the next will under sufficiently large sampling intervals become random, with directional persistence decreasing with increasing sampling interval. The mechanistic relationships explaining the moment-to-moment behaviour (reflecting more or less deterministic “movement rules”) are then hidden at finer temporal scales.

On the other hand, the LFF hypothesis defines BM in a more direct and mechanistic manner, linked to actual search behaviour. The model explicitly describes both BM and LW as alternative modes of movement (movement rules), while BM is not considered a consequence of fix sampling at coarse temporal scales as described above. In other words, when an animal is foraging in BM mode, it is assumed to perform successive steps in more or less random direction with a Gaussian-distributed step length at fine temporal resolutions. When in LW mode, the successive directions are still random, but the step length distribution is scale-free (power law compliant) (Figure 1A).

Recently, LW-like movement has been statistically identified in a wide range of species, covering animals as diverse as jellyfish [12], sharks and albatrosses. Humphries *et al.*[6, 7] and Sims *et al*. [8] have recently provided the most extensive empirical tests on the LFF hypothesis, from respectively analysing movement paths of predatory fish (14 species and 55 individuals), albatrosses (*Diomedea exulans* and *Thalassarche melanophrys*) and white shark *Carcharodon carcharias* (4 individuals). In general, LW was found in resource poor environments, while BM was verified in more resource rich environments in compliance with the LFF hypothesis.

Question then arises whether the two kinds of search patterns – BM and LW (and their intermediates) – are the result of behavioural mode switching (the LFF hypothesis) or a consequence of the sampling regime of fixes. For example, if LW was the only foraging rule, a BM pattern might arise in resource rich environments as a consequence of the higher frequency of interrupts of the otherwise long steps in a LW (leading to so-called “truncated LW”) [13, 14], while LW characteristics of the path is still maintained at finer temporal resolutions than the average interrupt interval. From this alternative hypothesis the observed transitions between BM and LW would be an artifact from the different interrupt frequencies on directional persistence of movement paths in different resource regimes. This aspect has not received a proper attention in the context of the LFF hypothesis. Further, there is also another aspect missing in the model: explicit memory implementation. The LFF hypothesis constrains movement to strictly opportunistic search for food, since both LW and BM depends on a premise of memory-less movement under varying degree of scale-free space use. “Memory-less” in this context means that a BM or LW path is self-crossing by chance only, not by intentional returns to a previously favourable location. Memory-less movement may be a feasible assumption for foraging in environments with truly stochastic dispersion of resources in both space and time, but otherwise memory effects in the form of non-random returns to previously visited patches (site fidelity) may lead to more complex space use than the BM-LW continuum is expressing. These two LFF model issues, effects from temporal sampling scale and potential influence from memory-enhanced movement, will be addressed below. In particular, we show by simulations how increased site fidelity may be misinterpreted as BM under conditions where a site fidelity-influenced path is sampled at coarser intervals than the average interval between directed returns to a previous location. Tests of the LFF hypothesis where this aspect is not accounted for are consequently inconclusive. We also supplement previous empirical tests that illustrate how the alternative movement model can be tested on real data, and we argue that this *a priori* approach should forerun tests of the LFF hypothesis itself.

### From Markovian-compliant to memory-influenced space use

Memory-less movement as it is defined by the BM-LW mode switching under the LFF hypothesis implies a Markovian process at the fine-grained mechanistic/behavioural scales. Under this assumption the animal is considering where to move next independently of the path that brought it to its present location. The next move (*i.e.*, the spatial displacement during a small time interval) during a foraging bout is then determined by local conditions within the individual’s present sensory range. In the case of LW, directional persistence from one moment to the next may occasionally be maintained for a longer period than for a BM. However, the persistence depends successively on the foregoing moment’s path direction and not on the older parts of the path [4, 13–15]. Since – under the Markov assumption – the historic path that brought the animal to its present location does not influence its decision where to move next, successive moves are independent. In other words, in a tactical manner the animal decides its next direction and speed based on the dynamic interplay between local environmental conditions and the individual’s internal state; e.g., hungry or not, and (according to the LFF hypothesis) whether its foraging mode under the current conditions is tuned towards LW or BM.

The Markov condition seems to contrast with what we know about actual space use by vertebrates in general [16, 17] and fishes in particular [18, 19], which both have a cognitive capacity to recall historic information in a spatially explicit manner and executing a mixture of shorter-term tactical and longer-term strategic moves [20, 21]. A strategic move may be a decision to return to a previously visited location, based on a “memory map” of these locations and their respective properties. The taxonomic groups that have been favoured for LFF modelling are the ones that have been solidly verified to utilize a memory map under ecological conditions that favour site fidelity; i.e., the individual may return to previously visited patches in a manner which is not just random self-crossing of its path. Consequently, we propose that the process framework for the LFF model needs a generalization in the direction of memory implementation. This may be achieved by extending the Markov process framework to a two-dimensional continuum along the orthogonal axes 1) the degree of scaling (represented by BM towards LW; the Markov dimension) and 2) the degree of long term memory [22, 23]. In this manner, the standard Markov framework is embedded as the special condition where memory influence is sufficiently narrowed to be ignored both in model designs and in analyses of movement data (Additional file 1 summarizes an alternative model [24] where long term memory has been implemented within a Markovian model architecture).

Occasional directed return steps towards familiar sites; i.e., memory dependent site fidelity, may provide a potential for increased foraging efficiency where resources are abundant and relatively predictable [25–28]. Return events lead to constraint on space use relative to a purely opportunistic search, where space use is quantified as for example the polygon covering movement path over a given period of time. Hence, we propose that a scale-free movement model with different strength of site fidelity (difference in probability of a return event during a given time interval) under different resource conditions represents an alternative explanation for the LFF model’s mechanistic, modal shift between LW and BM (large and small diffusion rate) as a response to varying resource abundance. The scale-free aspect of this model extends the Markovian-type LW and memory-enhanced Markovian-formulated stochastic models, since both tactical and strategic goals are assumed to be executed simultaneously (see Discussion for an elaborated explanation), but with reduced interference between goals at different scales owing to difference in temporal resolution between tactics and strategy (Figure 1B).

By aid of simulations of this alternative model we demonstrate an apparently similar shift in LW towards BM as under the LFF hypothesis’ modal shift, but where the transition appear from scale-free movement spanning low towards high ratio of return steps relative to ordinary search steps; i.e., increased strength of site fidelity. Next we show how the site fidelity response to different resource conditions may be separated and quantified from analysis of GPS data. Further, we apply a previously proposed procedure [14] to differentiate between multi-layered scale-specific movement (Lévy walk look-alike “composite Brownian motion”) and true scale-free movement on a set of ca 50,000 hourly GPS fixes of 18 female red deer, *Cervus elaphus*.

## Results

### Simulations

We use the Multi-scaled Random Walk (MRW) model [10, 14, 23, 29] as an approach to implement scale-free search in combination with varying degree of memory-dependent site fidelity (see Methods and Additional file 1). Three time scales are defined: the implicit interval between successive displacements in simulations (*t*), the average return interval to a previous location (*t*_ret_), and the observation interval on the movement path (*t*_obs_). The latter represents GPS locations in real data, and is applied to study the effect from varying *ρ* = *t*_ret_/*t*_obs_ (relative strength of site fidelity for a given *t*_obs_).

#### The relationship between return steps and fix sampling time schedule

Average return interval *t*_ret_ is inversely related to intensity of site fidelity. Consequently, ratio *ρ* = t_ret_/t_obs_ is critical when testing for influence of memory. MRW that is sampled under condition *ρ*>> 1, whether due to relatively large *t*_ret_ or small *t*_obs_, may appear confusingly similar to memory-less LW [10, 23], hereby termed pseudo-LW since the process is memory-dependent but its effect is undetectable at the actual magnitude of *ρ*. The influence of *ρ* on the observed pattern is illustrated by the simulated series of fixes from two scenarios under the Markovian framework and four under the non-Markovian framework (using infinite memory; defined as return targets chosen among any previous location during the given simulation). The respective sets of fixes are sampled at observational scale *t*_obs_ = 10^3^ larger than the time increment for simulations, *t*.

**Figure 2:**
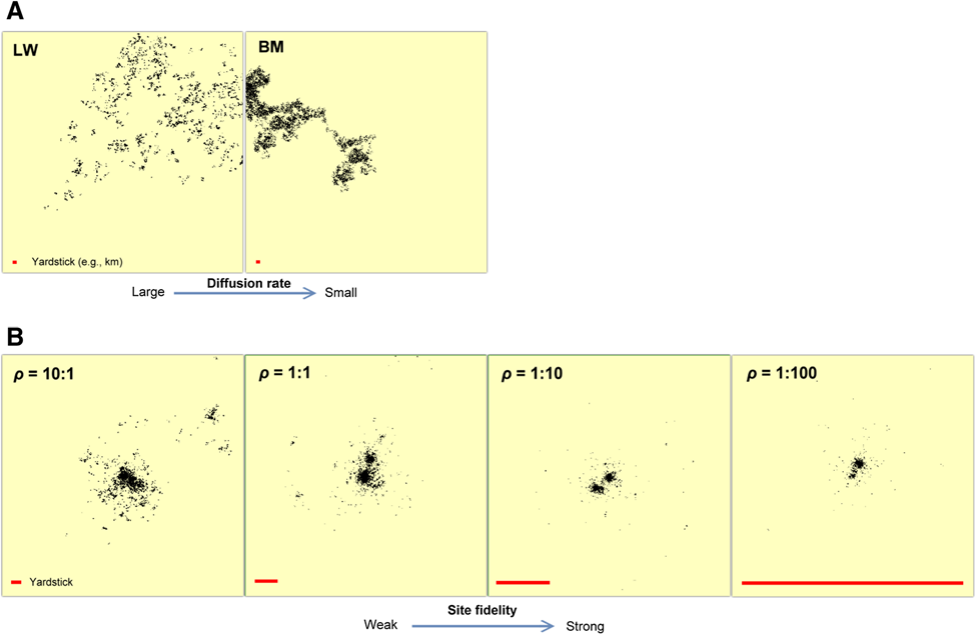
**The LFF hypothesis and the MRW-based variant both postulate a transition towards constrained area use with increased resource density.** While LFF describes this constraint by relatively smaller “diffusion rate” from a transition towards BM, MRW describes this constraint by stronger site fidelity relative to a given sampling scale under infinite or constrained memory horizon. The memory horizon regards how far back in time the animal choose previous locations for return steps (infinite horizon means that all previous locations are considered). **(A)** Spatial fixes from MRW with very narrow memory horizon (LW-like, left display) and a correlated random walk emerging as BM by sampling fixes at t_obs_ = 10^3^ (right display). **(B)** Four scenarios under MRW and infinite memory horizon, where frequency of return steps is increasing by a factor of 10 per display from left towards right. The embedded red line in the lower part of each panel shows the spatial scale (for example, 1 km) for the respective arenas. For a given accumulated movement length (total path), BM covers a smaller area than a LW. Similarly, MRW with a small return interval on average covers a smaller area than under condition of a larger return interval.

Under the Markov condition (Figure 2A), a BM covers less space than LW under a given movement speed at resolution *t* – the central property that supports the idea of adaptive switching of movement mode under the LFF hypothesis (constant speed is assumed at micro-scales <<*t*). The non-Markovian analogue to the transition from LW to BM is expressed in Figure 2B: scale-free MRW with varying strength of site fidelity but assumed constant movement speed at resolution <<*t*. Space use is in this case narrowed from successively smaller *t*_ret_, as observed from a constant *t*_obs_. For example, the arena to the right in Figure 2B, embedding the scatter of fixes under condition *t*_ret_ = 10*t* [*ρ* = *t*_ret_/*t*_obs_ = (10*t*/1,000*t*) = 0.01], has only 6.25% linear range compared to the left-hand arena [*ρ* = (10,000*t*/1,000*t*) = 10]. Thus, two processes, based on two different frameworks, may explain more constrained space use in resource-rich environments.

#### Step length distribution under a memory-enhanced process framework

**Figure 3:**
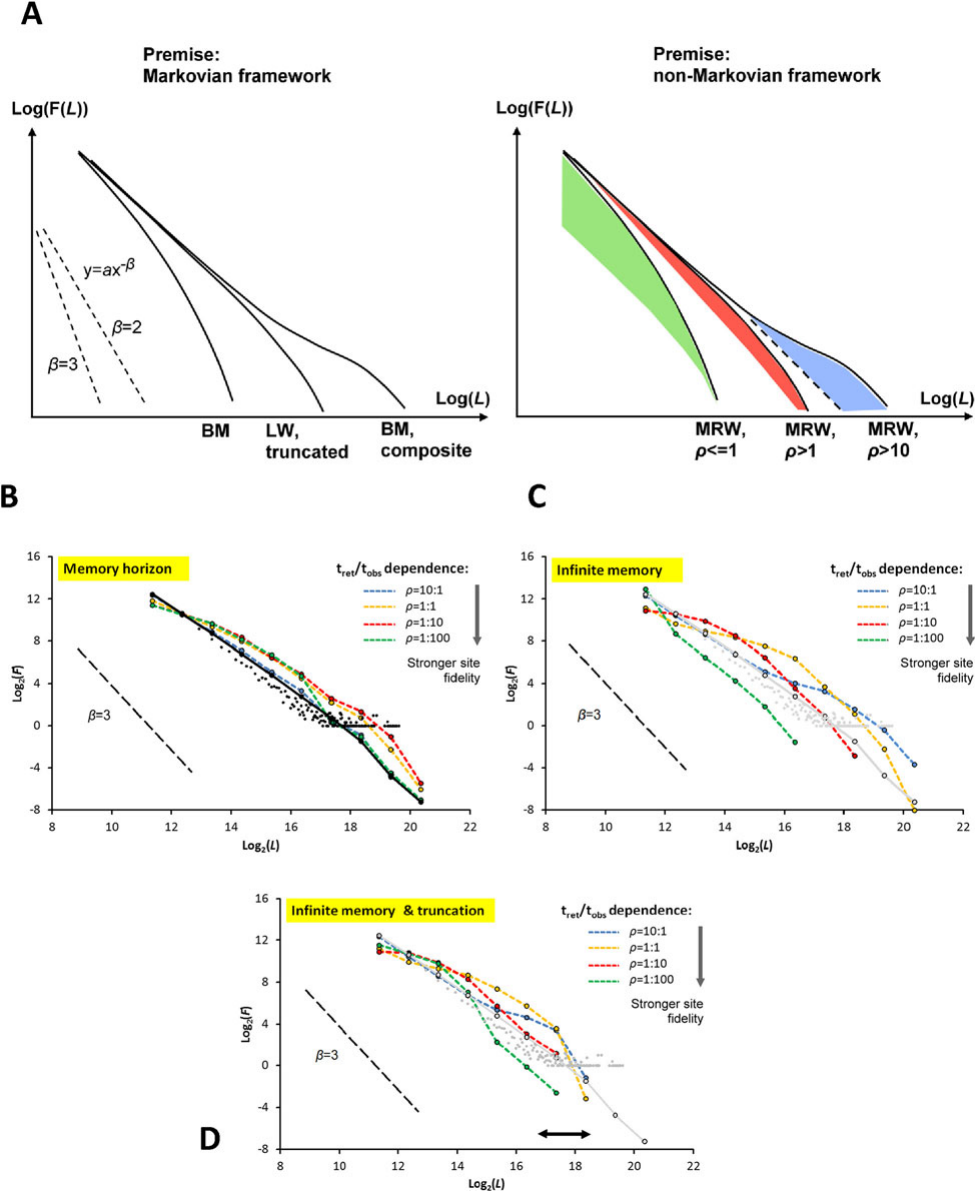
**Depending on the process premise for interpretation of step length distributions, patterns showing** ***β*** **> 3 may either verify BM under the Markov premise or MRW with** ***ρ*** **≪ 1 under the non-Markovian model premise.** Pane **(A)** illustrates this principle conceptually for three typical variants of the F(*L*) function under log-transformation of both axes. The coloured areas indicate how the influence from return steps may typically inflate F(*L*_*j*_) under three strengths of the site fidelity ratio *ρ* = t_ret_/t_obs_. The dotted line for *ρ* > 1 indicates the expectation either from very large *ρ* (leading to pseudo-LW), or from MRW under limited memory horizon (approaching the Markov condition, and thus expressing LW compliant movement). Panes **(B-D)** shows the result from simulations of MRW with *β* = 2, verifying that the observed distribution of step lengths depends on the ratio *ρ*; the individual’s return interval t_ret_ relative to the interval for fix collection t_obs_. **(B)** *ρ* = 10 and narrow memory horizon: estimated *β* is close to the simulation condition’s true *β* = 2. Under condition of *ρ* = 10 and memory horizon increased to 10,000 increments (blue line) a LW-like pattern with *β* ≈ 2 is still apparent but becoming more truncated under reduced t_ret_ (*ρ* < =1; yellow, red and green lines). **(C)** Infinite memory: increased shape-shift effect from reducing *ρ*, with plots appearing as truncated LW or BM-like (*β* close to 3). **(D)** Adding physical constraint on step length at spatial scale of the black arrow (see Methods) shows a similar pattern as in **(C)**.

Studying the fix samples in Figure 2B (non-Markovian processing conditions) under a premise of a Markov process may lead to erroneous results and an impression of a paradox unless the standard mechanistic model assumption (modal switching between BM and LW) is replaced by an analysis with both sampling interval and site fidelity accounted for. For example, the distribution of step lengths F(*L*_*j*_) (collected from the series in Figure 2B with supplementary variants) may typically tend to give a wrong impression of truncated LW – where the largest steps are under-represented – or a BM (slope parameter *β* >≈3). Stronger BM compliance apparently emerges with decreasing *ρ*, which is thereby influencing F(*L*) with an influence which the LW-BM continuum does not account for Figure 3 illustrates this issue conceptually (Figure 3A) and from simulations of various scenarios of memory horizon and intensity of site fidelity (Figure 3B-D; the respective plot series represent average distributions from eight replicates, in order to average out series-specific patterns). The memory horizon regards how far back in time the animal choose previous locations for return steps. The conditions *ρ* ≤ 10, from varying *t*_ret_ and keeping *t*_obs_ constant, are selected to show a temporal scale range where the site fidelity is particularly influential on the results.

For *ρ* = 10 and a narrow memory horizon of 10 time increments (Figure 3B) the pattern resembles the expectation from LW. This “reference condition” is represented both by arithmetic bin (black dots, showing some integer effect in the tail) and by log-binning [30] (black dots with connecting lines). Increasing the memory capacity to 10,000 time increments leads to some influence on the distribution under variable *ρ* (coloured series in Figure 3B), but the site fidelity effect is most noticeable under the infinite memory condition. The effect appears as inflated F(*L*_*j*_) over a sub-range of *j* (coloured series in Figure 3C-D). For example, for *ρ* = 10 this influence is located towards the extreme tail of the distribution, leading to a hump-like “hockey stick” pattern [14]. Additional file 1 shows how the hockey stick is almost invisible at *ρ* = 100, illustrating pseudo-LW variant of MRW for very large *ρ*.

As *ρ* is reduced by reducing *t*_ret_, the influence on F(*L*_*j*_) plots from site fidelity gradually shifts towards smaller *j* in a wave-like manner: the distribution shape-shifts as a consequence of sensitivity to *t*_ret_ relative to observer- defined *t*_obs_. Depending on choice of *t*_obs_ one gets an impression of a truncated LW as *ρ* is approaching 1, and then the impression of a BM (*β* >≈3) as the ratio is further reduced to *ρ* < 1. Hence, observing BM compliance from a F(*L*_*j*_) distribution based on real GPS data may either represent true BM-like movement or – if site fidelity is involved – a ratio between *t*_ret_ and *t*_obs_ which masks the underlying power law scaling under the given foraging conditions and average movement speed. Site fidelity may have been too strong (*t*_ret_ too small) to allow for observation of the scale-free movement component of foraging at the chosen *t*_obs_ for fix collection. However, deviations from power law as exemplified in Figure 3 may also result from other causes than memory-based site fidelity; e.g., a truncated LW due to a constraint on the appearance of very long steps (see description in Additional file 1).

#### Differentiating the Markovian from the non-Markovian framework

**Figure 4:**
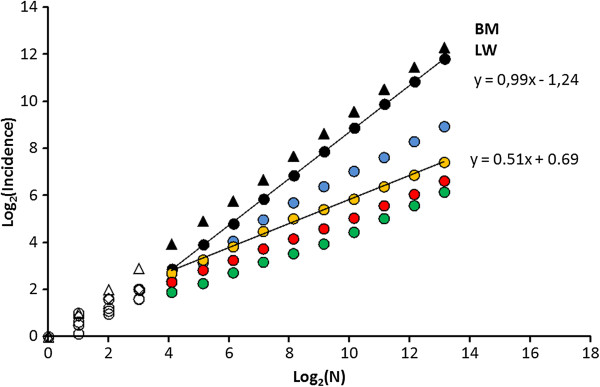
**The spatially explicit perspective of space use** ***I*** **(N)under variable** ***ρ*** **(same data as in Figure** 2 **) and constant grid resolution.** Both LW and BM confirms regression slope z ≈ 1 in double-log plot of *I*(N). Increased influence from return steps leads to *z* ≈ 0.5 for *ρ* 1 (colour references for respective *ρ* as in Figure 3), which is expected from *β* = 2 and infinite memory. The transition zone from auto-correlated towards non-auto-correlated spatial relocations (*ρ* ≈ 1; blue circles) leads to an intermediate power exponent. When analyzing real data in a similar manner, grid resolution should be varied to fit the scale where intercept *c* ≈ 1 [log(*c*) = 0)]. This unit pixel size then equals the movement’s characteristic spatial scale [23].

Fortunately the confusing patterns in Figure 3 – LW, BM or a transition between LW and BM (truncated LW) in compliance with a Markovian process framework, or MRW in compliance with a non-Markovian variant – may reach further clarification by applying a spatially explicit study of fix dispersion (Figure 4). Both BM and LW are discarded as an explanation for the pattern in Figure 3 owing to verification of site fidelity. Only MRW complies both with power exponent *z* <<1 in *I*(N) = *c*N^*z*^ (implying memory-based returns; see Methods) and scale-free space use (owing to the constancy of *z*; i.e., a power law relationship, over a large range of N). Figure 4 shows the transition from true BM and LW from approximately memory-less MRW (short memory horizon) towards a memory- influenced scale free process (MRW with infinite memory). As expected, the slope *z* in a log-log plot of *I*(N) is reduced in the transition zone between memory-less and infinite memory and then stabilizes despite further decrease of *ρ*, from keeping *t*_obs_ constant and reducing *t*_ret_. In other words, *z* is thereby shown to be independent of the spatially explicit site fidelity strength, as expressed by *c*. For the three conditions of infinite memory and *ρ*< = 1, the plots illustrate that a reduction in *ρ* leads to a reduction in *c* (the intercept with the y-axis). For example, the line y = 0.51x + 0.69 (for the condition *ρ* = 1), gives *c* = 2^0.69^ = 1.61 grid cells at the *a priori* chosen grid resolution. This implies that the “characteristic average scale”, expressing strength of site fidelity in this case is 1/1.61 = 0.62 grid cells.

### Analyses of GPS fixes

Four results supported the non-Markovian framework over the Markovian framework from the red deer data.

**Figure 5:**
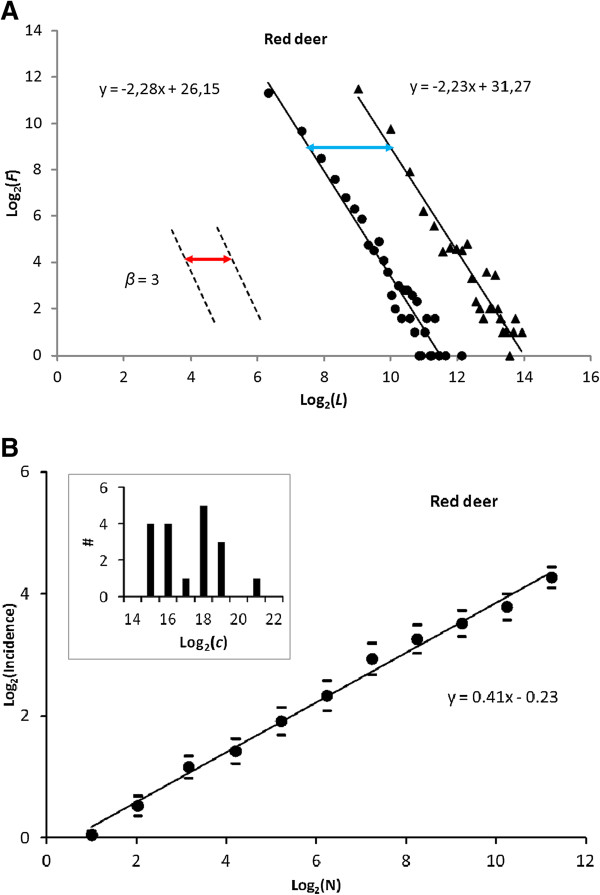
**Space use by red deer in Norway recorded by aid of GPS, averaged over 18 individuals. (A)** Lagrangian view; measuring parallel shift from increasing t_obs_ by a factor of 10, from 1 hr to 10 hr. The similar magnitude of *β* and the magnitude of the parallel shift (blue arrow) both confirm scale-free movement, which may be LW-like (Markov-compliant) or MRW-like (memory-influenced). **(B)** Eulerian view; incidence log(*I*) as a function of sample size of fixes log(N) supports MRW over LW, since *z* = 0.41 <<1 and stable (implying a scale-free kind of site fidelity) over a substantial range of N. The plots show the average *I*(N) for 18 individuals (+/- 1 standard error), where each individual’s characteristic spatial scale parameter *c* has been normalized to *c* ≈ 1 by respective grid scale adjustments (Additional file 1). This normalization does not need to be exact; it is trivial to estimate *c* = 1 [log(*c*) = 0] from a somewhat smaller or larger value given by the regression. The distribution of the actual *c*-estimates (magnitudes in m^2^) is shown in the inset.

First, the parallel shift of F(*L*_*j*_) (see Methods) of the pooled set of step lengths by changing *t*_obs_ by factor of 10 was 5.7 (blue arrow in Figure 5A), which deviated only 7.5% from the theoretical expectation 6.16 [rms(X) ≈ X^1/(2.26–1)^ = X^0.79^ = 6.16], based on the estimated *β* = 2.26. This diffusive rate is 80% larger than the expectation for LW-like composite BM; with *β* > =3 [rms(X) ≈ X^1/(3–1)^ = X^0.5^ = 3.16]. This result confirms super-diffusion as expected from scale-free movement, which may be caused both by MRW observed from *ρ* ≫ 1 and by LW.

Second, the approximate constancy of *β* over a substantial range of *L*_*j*_ even while increasing *t*_obs_ (reducing *ρ*) by a factor of 10 verified that red deer were capable of moving in a truly scale-free manner rather than composite BM [14, 15]. Hence, the Lévy walk look-alike condition composite BM [31] was rejected as an explanation for the power law compliance.

Third, analysis of F(*L*_*j*_) for each individual revealed that by changing *t*_obs_ from 1 h to 10 h in all cases resulted in a larger *β*, for some individuals even leading to *β* >=3 as a consequence of this decrease of *ρ*. The respective average *β* was 1.91 for t_obs_ = 1 h and 2.45 for *t*_obs_ = 10 h (paired t- test, 2-sided: t = -6.39, 16 d.f., p < 0.001; table A1, Additional file 1). This result – with reference to the issue that was illustrated by simulation results in Figure 3 – would under a Markov assumption be expected from a truncated LW situation [32, 33] and under non-Markovian assumption from MRW-compliant movement [14, 15]. In the latter case a larger *β* is expected from a decreased *ρ* as *ρ* ≈ 1 is approached from a larger ratio. The observed increase in *β* for individual series was concealed in the pooled set of steps in Figure 5A, where the truncation of the longest step sizes for some individuals was counteracted by other series showing “hockey stick” from hypothesized return step effect in the extreme tail of the distribution. Further, at the individual level bin size was defined for each series separately, rather than using a common bin size as in the pooled set (Additional file 1). This contributed to an improved estimate of the effect on *β* from changing *t*_obs_. The pooled analysis for Figure 5A was chosen to focus on the over-all estimate of the parallel shift.

Fourth, the complementary *I*(N) analysis, modified relative to Gautestad *et al*. [22] owing to approximate normalization of *c* for each series, showed a power law exponent 0 ≪ *z* ≪ 1 (Figure 5B). In compliance with the results in Gautestad *et al*. [22], the *I*(N) analysis was necessary as a supplement to the distribution F(*L*_*j*_) to differentiate Markovian versus non-Markovian movement.

However, here the result is sharpened by the normalization procedure for choosing grid resolution. The present result extends the *I*(N) result in Gautestad *et al*. [22] by confirming a wide inter-individual range of *c* from 0.02 km^2^ to 1.84 km^2^ (inset in Figure 5B), with a mean of 0.24 km^2^. Under the premise of the present model framework, this wide range of *c* is hypothesized to correlate with local conditions, for example average resource levels within the area covered by the respective individuals over the given GPS sampling period. As elaborated on in Discussion, *c* is a composite function of movement speed and strength of site fidelity.

## Discussion

### Empirical LFF tests are inconclusive

The LFF hypothesis predicts a more area-constrained movement from a behavioural transition from LW towards BM as the general resource level improves. With respect to energy gain from this assumed adaptive switching, Humphries et al. [7] found that total prey masses captured by wandering (*Diomedea exulans*) and black-browed albatrosses (*Thalassarche melanophrys*) during Lévy movements was similar to energy yields by Brownian movements in more resource rich habitats. In other words, the energy intake per unit time was fairly similar over the observed resource gradient, despite the high contrast between low resource conditions in open sea and high resource level in a shallower and more productive environment. Hence, under a premise of a Markov compliant process, a modal shift between scale-free LW and scale-specific BM apparently represents a positive energy value for the animal in comparison with a single-mode kind of movement. However, we here argue that also memory dependent site fidelity may provide a potential for increased foraging efficiency where resources are abundant and relatively predictable [25–28]. Thus, one may ask if a BM-resembling pattern from analysis of the step length distribution in empirical tests of the LFF hypothesis may actually have been pseudo-BM from memory-dependent site fidelity. Data analysis may have been performed under condition of a small ratio *ρ* between return events (at scale *t*_ret_) at the chosen observation interval (scale *t*_obs_). For example, Sims *et al*. [8] refer to site fidelity for some of the individuals that were subject to testing for LW-BM compliance, but an alternative process framework to the Markovian was not discussed. A transition towards BM-like distribution of step lengths, F(*L*_*j*_) may – according to our alternative hypothesis – be a statistical artifact from higher intensity of site fidelity without adjusting the GPS sampling frequency accordingly by narrowing of the fix sampling interval.

Interestingly, this kind of transition between exponential and power law distribution as a result of difference in site fidelity strength may have been documented by an analysis of GPS data of Balearic shearwaters (*Puffinus mauretanicus*) and Cory’s shearwaters (*Calonectris diomedea*) in the northwestern Mediterranean [34]. The results showed BM (*i.e.,* exponential distribution) in areas with fishing boat activity – which caused temporally improved local resource level for the seabirds – and LW-compliant distribution in absence of fishery (power law, with *β* ≈ 2). While the power law was generally found over a large time scale (*t*_obs_ in the range from 1 h to 12 h), the result from periods of trawler activity showed power law for the smallest *t*_obs_ of 1–3 h and exponential at larger *t*_obs_. In other words, this result may be seen as indicative of a MRW-like foraging by the seabirds, where *t*_ret_ is decreased (site fidelity is strengthened) in localities with fishery discards. As outlined above, a transition from exponential towards power law distribution of step lengths is expected if *ρ* = *t*_ret_/*t*_obs_ is increased by decreasing the denominator *t*_obs_ sufficiently to get *ρ*>> 1, as when reducing sampling of seabird fixes towards intervals of 1–3 h from larger intervals). However, the actual GPS results were taken as support for an alternative hypothesis; space use in compliance with a Markovian “continuous time random walk” (CTRW) model, where site fidelity is expressed differently (see below).

To understand and test for the *ρ*-effect, a statistical-mechanical perspective on memory-enhanced movement is required. A statistical-mechanical representation is achieved by sub-sampling every n’th relocation along a micro-resolved path and n is large. Further, in this approach one is studying specific patterns that emerge from a large sample of relocations, for example the slope in the distribution of step lengths. In Figure 1 and Figure 3 the respective steps that collectively produce the y-value for each of the bin intervals on the x-axis are collected from various times and places during the total period of sampling. In over-all (i.e.; statistical-mechanical) terms it is the large-sample property that provides the functional form and the power law slope parameter, given that we put aside both mechanistic details (by coarse-graining to level *t* and *t*_obs_) and also ignore details about each of the respective locations and step lengths contributing to the magnitude of each bin of the distribution. Below we discuss two main aspects in the context of distinguishing between LFF and the alternative model; the qualitative distinction between a Markovian and non-Markovian kind of space use, and why a statistical-mechanical level of analysis is required to interpret the results in Figures 3, 4 and 5.

### The qualitative differences between memory-less and memory-influenced search

Contrary to behavioural mode-shifting between scale-free LW and scale-specific BM under the Markov framework of LFF, MRW implies various strength of site fidelity (represented by frequency of return events) in combination with a scale-free kind of movement (represented by a Lévy walk in our simulations) along the entire resource gradient. However, a change in intensity of site fidelity is assumed to vary as a function of environmental conditions: stronger site fidelity is assumed in resource-rich and relatively predictable habitats, which was here modelled as a higher frequency of strategic return steps. As for the LFF hypothesis, we assume here (for simplicity) a positive correlation between resource abundance and predictability also for our memory-extended variant of the LFF hypothesis, but this premise may be relaxed in future model refinements.

The ratio between average return interval and observation interval, *ρ*, is an example of a statistical-mechanical quantity which captures both the essence of the site fidelity strength (the numerator) and the influence of various level of temporal coarse-graining (the denominator).

Obviously, this ratio makes no sense in a memory-less BM-LW context, since the numerator is always zero or not applicable at statistical-mechanical resolutions. When the return frequency in MRW simulations approaches zero (*ρ* ≫ 1 from large *t*_ret_ relative to *t*_obs_) under condition of assumed low and locally unpredictable resource abundance in the present model extension, a pseudo-LW pattern is expected. Under this condition, memory influence will only be apparent under very long-lasting studies. In shorter studies return steps are less likely to be observed. On the other hand, if *ρ* < 1, a pseudo-BM is expected.

### Getting an indication of processing framework from F(*L*_*j*_) alone

In Results we argued that a spatially explicit analysis (Figure 4) was necessary to differentiate a Markov-based scale-free process from the non-Markovian counterpart. However, with reference to Figure 3, these variants may be at least indicatively distinguishable also from a study of the step length distribution F(*Lj*) alone, by testing for the predicted *ρ*-effect under conditions where a BM-like pattern is found in a data set. The arguments go as follows, with respect to the premise of MRW: if the observation interval *t*_obs_ is reduced, *ρ* = *t*_ret_/*t*_obs_ is increased from reducing the denominator for a given numerator (new and higher-frequency sampling may be necessary to achieve this, if the complete set of fixes was applied for the initial analysis). If such narrowing of *t*_obs_ is sufficiently strong, the MRW model predicts that a BM-like step distribution F(*Lj*) should appear more power law-like as a result of increased *ρ* from reduced *t*_obs_.

For example, if an individual’s path in a resource-rich environment under F(*L*_*j*_) analysis appear BM-like from *t*_obs_ = 1 h, but LW-like from *t*_obs_ = 5 min, the non-Markov framework is supported and the Markov framework weakened. The MRW model predicts re-emergence of the power law pattern observed from F(*L*_*j*_) regardless of environmental conditions, given that *t*_obs_ is reduced accordingly to avoid the observer-effect from too small *ρ* owing to a smaller return interval *t*_ret_.

In contrast, the LFF model implicitly predicts a transition from Poisson-distributed BM steps towards a correlated random walk-like path with Gaussian-distributed steps if *t*_obs_ is sufficiently decreased (revealing stronger directional persistence between successive displacements and thus a “smoother” path than BM for a similar sample size of fixes) [5, 11, 35]. The transition from a Poisson to a Gaussian step length distribution emerge at fine path resolutions where each inter-fix distance corresponds well with relatively linear path segments and thus reflects true movement speed *L*’/*t*_obs_ for respective steps of length *L*’. Correlated random walk, like BM (whether sampled in the Poisson or Gauss scale regime), is not power law-compliant with respect to F(*L*); see Additional file 1.

A MRW path might also show transition towards a pattern that may resemble a correlated random walk-like path (with Gaussian distribution of step lengths) at very high-frequency fix sampling imagined at temporal scales <<t. However, while a true BM leads to transition towards correlated random walk and a Gaussian distribution directly during such “over-sampling” of a path, an under-sampled MRW [leading to a BM look-alike path from the perspective of the distribution F(*L*_*j*_) from large *t*_obs_] will show a transition towards power law pattern under reduced sampling interval *t*_obs_, before showing the over-sampling effect due to entering the scale range for a “smooth” and correlated random walk-like pattern if *t*_obs_ is reduced too strongly (*t*_obs_ <<*t*). In other words, BM from a Markov process lacks the intermediate scale range of power law-distributed steps when path resolution is increased by reducing *t*_obs_.

This approach was applied here on the individual red deer GPS data (Additional file 1). By changing *t*_obs_ from 1 h to 10 h (decreasing *ρ* from increasing *t*_obs_) in all cases resulted in a larger *β*, for some individuals even leading to *β* >=3, which from a Markovian BM-LW perspective would indicate a transition from LW towards BM. Conversely, as outlined above, a transition from a BM pattern at scale *t*_obs_ = 10 h towards a LW-like pattern at scale *t*_obs_ = 1 h supports a non-Markovian space use like MRW. Under this class of statistical mechanics the transition is predicted from the model.

However, the result is not conclusive, since a Markovian LW will always be subject to a larger or smaller degree of long-step truncation, which may hide the power law property of LW. A long step takes longer time to execute, and – consequently – such displacements are more prone to being prematurely terminated by various environmental influences (or limited by maximum movement speed). Sampling a path at *t*_obs_ larger than the average disturbance interval will lead to a BM pattern in the distribution of step lengths (with truncated LW in the transition). Sampling a MRW at *t*_obs_ > *t*_ret_ also leads to BM pattern [10, 14]. Hence, the *I*(N) analysis of fix dispersion is needed as a complementary analysis to differentiate between memory-less and memory-influenced space use (Figure 2B and Figure 5B).

On the other hand, one may argue that a LW is never expected in a resource-rich environment, owing to a relatively high frequency of interrupts on path directionality. Since the present red deer data actually show power law distribution of steps even at *t*_obs_ as large as 1–10 h (Figure 5A) while foraging in a resource-rich summer season environment (see also ref. [22]), it is reasonable to propose that a mixture of tactical and strategic goals – in compliance with a non-Markovian kind of movement – may explain the maintenance of long steps in the power law distribution of step lengths. A long step in progress may be “back-on-track” after a temporary goal has been taken care of [23], as explained in Figure 1B and in Additional file 1. Hence, to understand the emergence of a power law compliant step length distribution in a high-frequency disturbance environment a non-Markovian variant of movement is postulated for the MRW model. The following section summarizes this expansion of classical statistical mechanics [10, 23, 27, 29], while simultaneously elaborating on previously published descriptions in this regard.

### Memory-influenced foraging under the non-Markovian framework

The difference between a Markovian and non-Markovian implementation of memory in space use models is explained in more detail in Additional file 1_._ Strategy implies processes that embed future goals based on memory of conditions outside the individual’s present perceptual field and back in time. Strategy – according to the non-Markovian system description – is executed in parallel with tactical decisions, the latter representing responses to the immediate environment. Under the non-Markovian memory model, a short-term goal to move in one direction may be performed even if a longer term goal requires another direction. Thus, a “parallel processing” kind of strategy/tactics execution deviates qualitatively both from adaptive search under the Markov-compliant LW-BM continuum (memory-less search; as in LFF) and from a Markovian implementation of memory effects on foraging [24]. In the latter case the relative “weight” of the two goals always decides the next step direction in a moment-to-moment recalculation manner (Additional file 1).

According to the non-Markovian framework, a temporally coarse-grained goal (like reaching a specific site within a medium or longer term period) may be executed more or less un-influenced by shorter term goals that involve intermediate tactical responses [23] (Figure 1B). Without some insulation between goals at different temporal scales (i.e., conceptually at “different wavelengths”), where the coarser-scale goals put constraint on finer-scale goals, strategic goals would be constantly subject to interference from shorter term events. Indeed, in the empirical literature of hierarchical foraging decisions, it is well documented that different factors affect decisions at different scales [36]. Memory map utilization over a large time horizon leads to a tension between foraging tactics and strategy in the foraging process, as indirectly expressed in qualitative/structural terms by the concept of hierarchical landscape utilization [36]. Dynamically, a food patch may be inexplicably abandoned or ignored from a tactical perspective, but this choice may be explicable from a coarser strategic perspective [23, 37]. For example, if the individual in Figure 1B left the patch C’ while it apparently still offered non-utilized food resources, it might have been a consequence of running out of time to achieve the coarser-scale (longer term) goals B’ or A’. An animal deciding to move from summer to winter range may be termed a long term goal from a perspective of a space use study involving hourly fixes, while an animal deciding to reach a resting patch within a few hours may be termed a long term goal from the perspective of a fix series collected at minute-by-minute resolution.

MRW has previously been verified – at a statistical-mechanical level of analysis – to represent vertebrate space use more realistically than movement models from the Markovian framework [22, 38, 39]. The site fidelity effect as expressed by *ρ* was supported by the present results from red deer data, where *t*_obs_ was varied. Above we also referred to seabird foraging [34], which was compliant with expectations from the *ρ*-aspect of the MRW model. However, the seabird results were interpreted as support for an alternative model, based on continuous time random walk (CTRW).

### Site fidelity under the continuous time random walk model

CTRW describes a Markovian kind of stochastic movement under assumption of studying a movement path at statistical-mechanical scales, where the step length distribution and a waiting time distribution (“resting” between steps) describe mutually independent random variables. Interestingly, Bartumeus *et al.*[34] show how both distributions may be obtained from GPS data, and they formulate the waiting time distribution as expression of site fidelity (see also [40], where mathematical aspects of the spatial and temporal properties are further enhanced). In this model stronger site fidelity implies a smaller median staying time in superior foraging patches [34]. In terms of diffusion, the CTRW is expressing site fidelity by a power exponent (*i.e.,* a scale-free process), which quantifies how the mean square displacement is expected to grow as a function of time. In simple terms, consider how the area of a circle – with the path’s starting point at the circle’s origin and the moving object at the circle itself – is growing under weak and strong site fidelity. An exponent of 1 reflects normal diffusion (area growing proportionally with time). An exponent less than 1 implies strong site fidelity (expressed as scale-free “sub -diffusion”) while an exponent larger than 1 implies an opposite kind of space use, compliant with LW (“super-diffusion”). With respect to the seabird data, sub-diffusion and BM-like exponential distribution of step lengths was found in superior foraging areas (from presence of fishery discards), while super-diffusion with LW-like distribution of steps was found elsewhere. In other words, the model spans the same BM-LW continuum and Markovian architecture as under the LFF hypothesis. However, a modal shift from LW towards BM in the step distribution is accompanied by a scale-free waiting time function for inter-step pauses and respective spatial and temporal parameter intervals for the LW and the BM conditions.

CTRW under sub-diffusion may be considered a Markovian counterpart of the non-Markovian MRW model with respect to modelling site fidelity at statistical-mechanical temporal resolutions. Despite the temporal scale-free distribution of waiting times under CTRW (which consequently implies infinite memory), this kind of memory is not spatially explicit. There are no goal-oriented returns to previously known sites, as defined under MRW. Site fidelity under CTRW implies a continuum involving long term memory in mathematical terms in order to include a potential for very long waiting time events at a given location, but execution of this behaviour is strictly local and sequential. There is no parallel execution of independent goals at different temporal scales and no directed returns to locations visited in the past, as outlined for the MRW framework above.

Owing to the qualitative differences between CTRW-based and MRW-based implementation of site fidelity, it should be possible to distinguish between these two alternative hypotheses. A protocol for this is presented next.

### MRW and optimal foraging: a test procedure for differentiating Markovian from non-Markovian space use

Here we have implemented the MRW model into an optimal foraging context by hypothesizing scale free-movement with return events that take place less frequently (weaker site fidelity) in a resource-poor environment. Conversely, stronger site fidelity (under a given movement speed) implies a more constrained space use (Figure 2A). Specifically, the strength of site fidelity as a function of return frequency is here for the first time coupled to a specific parameter *ρ* in the *F(L*) approach (step length distribution), representing a complement to the parameter *c* in the spatially explicit approach (spatial scatter of fixes).

This prospect to differentiate the Markovian from the non-Markovian framework from a set of GPS fixes can be summarized as follows: (a) Use a sufficiently large GPS fix sampling interval (*t*_obs_) to avoid confounding effects from path oversampling; (b) from analysis of the step length distribution (Figure 1A), study the degree of power law compliance and magnitude of median step length at different magnitudes of *t*_obs_; and (c) by superimposing a virtual grid on the spatial scatter of fixes, study the number of grid cells that embed at least one fix (“incidence”, *I*) as a function of sample size of fixes (N) (Figure 1C). In particular, estimate the degree of power law compliance and magnitude of “grid area per fix” where grid area is represented by *I*, after normalizing the function *I*(N) by varying grid resolution. Normalization is achieved when the regression line in Figure 1C may be interpolated through the origin in the log-log plot.

Element (a) in the protocol is necessary to ensure pattern analysis at statistical-mechanical level, so that the three movement classes LW, BM and MRW can be differentiated with the advantage of averaging out fine-scale heterogeneity at the mechanistic micro-scales [11]. Element (b) is a method to quantify the diffusion rate over a scale range in order to differentiate true scale-free movement (LW and MRW) from the scale-specific look-alike movement type “composite BM” (a superposition of various BM modes) [14, 15]. Finally, element (c) is necessary to differentiate more clearly between the two process frameworks Markovian (including CTRW models) and non-Markovian space use in a statistical-mechanical sense; a varying strength of MRW-compliant site fidelity as opposed to a varying strength of diffusion under the LW-BM continuum [23]. As an extension of previously published aspects of MRW, we show here how the unit scale – represented by the log-log origin (0,0) after normalization of *I*(N) in Figure 1C – corresponds to varying intensity of site fidelity under the present space use model. For example, individuals may vary with respect to the magnitude of this unit scale and (with particular relevance for an optimal foraging context) one individual my show difference in this unit – the “site fidelity” parameter – under different environmental conditions. Thus, the site fidelity parameter *c* represents the non-Markovian framework analogue to the diffusion rate aspect (sub-and super-diffusion) under the Markovian framework.

While strong site fidelity under the referred CTRW model implies sub-diffusion with BM-like step length distribution, one should anyway expect *I*(N) = *c*N^*z*^ to show *z* ≈ 1. *I*(N), if sample size N is set proportional to the time period for GPS sampling, is an alternative to mean square deviation to quantify constrained space use and its scale-free properties [22, 23]. *z* ≈ 1 was verified for classic BM and LW in Figure 4, while MRW-based site fidelity leads to *z* ≈ 0.5. The latter emerges as a consequence of targeted returns to previous locations. Since CTRW lacks such targeted returns *I*(N) is expected to expand proportionally with time under this process regime. This should happen whether the expansion is slow (as under sub-diffusion) or fast (as under super-diffusion) and whether the process is scale-free in space and/or time or not. When the moving object departs from a given location, the probability of return is due to chance, with a probability similar to visiting any other location at a similar distance from the objects’ new location (whether previously visited or not). In simple terms, this property of sub-diffusion under a premise of absence of targeted returns leads to the expectation that *I*(N) should expand proportionally with N (where N is proportional with time), as was shown for BM and LW in Figure 4. Since the *I*(N) aspect has not been tested on the seabird data in [34], one cannot conclude at this point whether the site fidelity actually was CTRW compliant (Markovian) or MRW compliant (non-Markovian). However, the observed transition in favourable localities from exponential towards power law distribution of step lengths as a result of shortening the fix intervals to 1–3 h supports the MRW model (but with some uncertainty, as described in general terms in the section “Getting an indication of processing framework from *F*(*Lj*) alone” above).

As already explored in previous simulations of MRW [25], varying movement speed will– like a difference in the average return interval *t*_ret_ – also influence *c* in the space use function *I*(N) *= c*N^*z*^. A slower movement speed (e.g., measured as average m/s) relative to a given return rate was shown to reduce *c* in [25]. Hence, *c* may be considered a function *c*(*t*_ret_,*v*), where *v* represents the average speed within the given time span and area covered by the analysis of *I*(N). Accordingly, the respective estimates of *c* for red deer as shown in Figure 5B should be adjusted for varying movement speed between individuals under the respective environmental conditions prior to testing the site fidelity response (an adjustment function was presented in Gautestad and Mysterud [25]). For example, under condition of a similar resource level, one area may show a smaller *c* relative to another due to a more jagged terrain (like a stony hillside with many speed-influencing local obstacles). However, an estimate of average movement speed (sampled at the micro-scales where the path is relatively smooth) for the actual terrain and data sampling period could then be applied to adjust *c* prior to comparing site fidelity with a resource map. These variations in movement speed could be obtained from series of high-frequency GPS sampling (for red deer, *t*_obs_ <<1 h) in different environments. In this manner, these two components of *c* may be disentangled. For example, if relative movement speed is found not to correlate significantly with resource level but speed-adjusted *c* does, the memory component of site fidelity *ρ* is shown to be influenced by the resource level. Accordingly, the LFF hypothesis is weakened, since it depends explicitly on difference in diffusion rate (which is a function of both movement speed and whether movement is LW- or BM-like) but not on the *ρ* aspect of the space use parameter *c*. Other unexplored aspects of the spatially explicit space use parameter *c* and its Lagrangian complement *ρ* may also contribute to an improved estimate of site fidelity under different ecological conditions.

### MRW and the LW controversy

GPS data from vertebrate movement that are collected at a rate of for example one hour are likely satisfying a statistical-mechanical level. The results in Figure 3 illustrate how a statistical-mechanical approach in combination with an explicit implementation of long term memory effects may contribute to resolving some of the controversy surrounding whether some specific data set conforms to LW or BM [4, 8, 11, 41, 42]. In particular, a purely statistical approach by arguing for and against specific statistical methods [4, 43–45] will be insufficient if the effect from observational scale and influence from site fidelity are not both explicitly considered. As shown by the simulation results in Figure 3, two studies using data with different time intervals between positions (*t*_obs_) for the analysis would easily reach different conclusion with respect to LW and BM (under a premise of the Markovian framework), regardless of statistical procedure. These kinds of conflicting results would also hamper clarification with respect to BM/LW modal shift under the LFF hypothesis, the alternative MRW model based on site fidelity and other movement models that may be applied to study optimal foraging. In short, a focus on the underlying space use process with an explicit consideration of a potential for site fidelity effect and a consequential breach of a core premise for the LW-BM model continuum may turn out to be more clarifying than choosing between specific statistical protocols to differentiate between degrees of power law compliance in the distribution of step lengths. Even the highly appreciated Maximum Likelihood Estimation (MLE) method with Akaike weights [42] has weakness with respect to verifying power law compliance [45]. It is interesting that one of the main issues raised in this respect regards the “problematic” occasional over-representation of very long step lengths even relative to an ideal Lévy walk distribution, invoking the term “Lévy walk-like” search [8, 45]. This “hump” in the long tail part of the distribution has been hypothesized to emerge from some kind of environmental forcing [45]. However, here we have shown (Figure 3) that a similar hump – called a hockey stick – is in fact expected by default if MRW-compliant data are analysed within a specific range of the ratio between return events and observation interval.

### Modelling framework and the way forward

We have illustrated varying intensity of site fidelity by simulations of the MRW with constant movement speed and varying return interval t_ret_. We have paid specific attention to the effect of the ratio *ρ* = *t*_ret_/*t*_obs_ on the observed pattern of space use, where *t*_obs_ is the time interval between two successive relocations (in practice, the fix interval in a sample of GPS data). Further, we studied the feasibility of the non-Markovian framework of MRW relative to the Markov framework of LFF from extended analysis of GPS summer season data of female red deer. In Figure 2A the continuum between LW and MRW was described as a narrowed memory horizon for return moves. Implicitly, memory does not pay off in an unstable environment. Simulations of MRW have confirmed that the power law pattern of grid-calculated space use, *I*(N), may be maintained also in a temporally less stable environment, which may lead to a drifting spatial range for habitat utilization [27]. This variant of the MRW boundary conditions underscores that resource predictability does not necessarily correlate positively with resource abundance – an aspect that has not been explored in the context of LFF.

We have used three aspects of fix series to determine in qualitative terms which process framework is the most feasible for a given data set, and hence which set of premises and parameters that should be in focus for the ecological analyses. We promote a two-stage process, where the Markov assumption should not be accepted a priori but should be validated by the abovementioned procedure before decisions regarding the analyses of how movement and space use variation may be correlated with the actual resource (and other) conditions. Crucially, owing to the qualitative difference between foraging with and without memory map utilization, the statistical study design should be based on which framework that is actually supported by the data. For example, the site fidelity parameter that is central to the present development has no application under a Markovian approach. One should choose the Markov framework for the analysis if memory has been shown not to be an issue and conversely one should choose the memory-extended framework if site fidelity has been verified.

## Conclusion

What may appear as transitions between memory-less BM and LW in animal search may alternatively be a non-Markovian kind of scale-free and memory-influenced space use – represented in our simulations by MRW – studied under different ratio between average return intervals relative to the chosen observation interval for fix sampling.

## Methods

### Simulations

We use the Multi-scaled Random Walk (MRW) model [10, 14, 23, 29] as an approach to implement scale-free search in combination with varying degree of memory-dependent site fidelity. A constant time interval, *t*, is defined for successive steps. This time interval is assumed to be large enough to support the model condition of a random direction of successive step vectors. Hence, *t* is postulated to implicitly embed a series of “hidden” (un-observed) moves from unspecified mechanistic movement rules that are executed at finer time scales. This coarser level system definition – together with the accompanying pattern analysis based on a large sample of relocations – makes MRW statistical-mechanical. Thus, the model is in this respect similar to BM and LW, which are also statistical-mechanical models in strict terms (see Background and Discussion). However, MRW extends the BM-LW continuum by adding site fidelity from spatial memory influence. MRW describes LW-like exploratory “search” steps (movement component 1 of MRW, represented by power law distributed displacement lengths at temporal scale *t* with scaling exponent 1<*β*<3; see Figure 1A legend) and site fidelity in the form of occasional return steps (movement component 2, at average interval *t*_ret_, which is larger than *t*).

*β* = 2 is chosen by default, since this magnitude of the power law exponent leads to a balanced statistical mixture of many fine and fewer coarse-scaled strategic moves [29]. Further, the initial simulation studies that led to the LFF hypothesis showed optimal foraging results for *β* ≈ 2 where resources where scarce and unpredictably distributed. Larger *β* leads to a relative higher frequency of the smaller moves. Empirical studies of both marine and terrestrial vertebrates also typically show *β* in the range 1.5 < *β* <2.5 where power law compliance have been found.

Exploratory steps under MRW were simulated from standard LW procedure (Additional file 1), with occasional return steps towards randomly chosen previous locations at intervals *t*_ret_ every 10,000^th^, 1,000^th^, 100^th^ or 10^th^ time increment *t*, within a trailing time window of size 10 *t*, 10,000 *t* and infinite. The latter implies that targets for returns were chosen among all previous locations in a given series, regardless of the length of the simulation period. A narrowing of the trailing time window for potential return targets illustrates a transition from memory-enhanced MRW towards memory-less LW. The random return target condition may seem counter-intuitive, but is feasible under the statistical-mechanical premise of sufficiently large *t*: deterministic rules which may optimize the tactical choice of which location to return to are assumed to be executed by the individual at temporal resolutions much finer than *t*. In other words, MRW is expressing the stochastic aspects of actual space use that emerge from a mixture of opportunistic search and return events, when this process is studied at statistical-mechanical scales.

The change of *t*_ret_ describes a relative change of site fidelity intensity, and represents a process continuum: a hypothesized progression towards smaller ranging area as site fidelity increases (smaller *t*_ret_ on average), for example this can arise if moving along a hypothesized environmental gradient with increasing resource abundance. In addition to the system’s time resolutions *t* and *t*_ret_, a third and independent interval, *t*_obs_, is representing the temporal scale where successive locations are actually collected for analysis. These series of “fixes”, which represent GPS locations in real data, are in the present simulations collected at a fixed *t*_obs_ = 10^3^ *t* to study the effect from varying *ρ* = *t*_ret_/*t*_obs_ on the observed distribution of step lengths. The scale- free ratio *ρ* is introduced to define strength of site fidelity relative to the transition between spatially auto-correlated and non-autocorrelated fixes. *ρ* = 1 is expressing the temporal scale where there is approximately one return step on average per relocation. A smaller return interval *t*_ret_ (like a larger observation interval *t*_obs_) relative to *ρ* = 1 brings the series of fixes into the non-auto-correlated domain [46]: the distance between two consecutive fixes is then expected to be similar in magnitude as two randomly drawn fixes from the data set. *t*_obs_ of magnitude 10^3^ *t* allows for a large range of *ρ* both above and below 1, and for comparing strength of site fidelity across species. For example, *ρ* = 1 for small species (e.g. a mouse) movement may be reflecting a much smaller *t*_ret_ and *t*_obs_ than *ρ* = 1 for large species (e.g. a bear), partly owing to the large difference in movement speed (we elaborated on this aspect in Discussion).

After adjusting for difference in movement speed, *ρ* represents the relative site fidelity strength from the statistical-mechanical step distribution perspective, and contains both a behavioural influence (the numerator of the ratio) and an observer-dependent component (the denominator). “Site fidelity strength” is here defined also intra-specifically since *ρ* from a variable *t*_ret_ and constant *t*_obs_ may be assumed to vary over time and space, as will be explored in the simulations. All simulations are performed under an assumption of a constant movement speed at micro-grained path resolutions, much finer than simulation scale *t*, in order to study the *t*_ret_ aspect of site fidelity. Hence, difference in step lengths at scale *t* are assumed to emerge as a consequence of differences in frequency of directional change (“path jaggedne ss”) during respective fix intervals *t*. Stronger jaggedness means smaller net displacement during t. Additionally, series were also produced with physical constraint on step lengths (adding to the return step effect on step lengths at scale *t*_obs_). Further details of the simulations are given in Additional file 1.

### Red deer data and statistical methods

GPS data of 18 red deer females (ca 2000–2800 relocations pr. individual) were collected by hourly spatial fixes during the summer season in a study area in Sogn og Fjordane county at the western part of southern Norway. These individuals have previously been verified to comply with a MRW-like movement, from a combination of scale-free and memory-influenced space use [22]. The same data are here subject to extended tests of the MRW compliance relative to Gautestad *et al.*[22], from two statistical protocols. These extensions allows for (a) differentiating between a true scale-free process from look-alike processes (composite scale-specific BM versus scale-free LW or MRW) [13, 14, 31]; and (b) quantifying strength of site fidelity from estimate of the specific site fidelity parameter under a premise of MRW-compliant space use.

(a) The “Parallel shift” method [11, 14] is applied to quantify the change in diff usion rate and to study the effect on the distribution of step lengths (F(*L*_*j*_)) (as it was defined in Figure 1A) by comparing plots at time intervals *t*_obs_ = 1 h and *t*_obs_ = 10 h (from re-sampling the original series). The parallel shift regards how the regression line for a distribution of steps (under log-transformed plotting) is parallel-shifted towards the right as a function of increased time between observations (*t*_obs_) in two sets of samples. Larger t_obs_ generally leads to larger steps, due to the larger time span between consecutive relocations. It also offers an opportunity to study the effect from decreasing *ρ* = *t*_ret_/*t*_obs_ from increasing *t*_obs_ rather than increasing the return interval *t*_ret_ (in the simulated data we decreased *ρ* by decreasing *t*_ret_, which is unknown in real GPS data). The two sample sizes should be of equal size to avoid a need to adjust for non-stationary variance of a power law distribution with exponent 1 < *β* < 3. A scale-free movement with *β* = 2 (whether the process is LW or MRW compliant) is expected to show a right-shift in proportion to *t*_obs_ (the parallel shift analysis applied on MRW data additionally depends on *ρ*> > 1). Doubling the sampling interval *t*_obs_ should approximately double the median step length. In contrast, simulations of a composite BM under a specific choice of parameters, or a LW/MRW that is made BM-like from choosing *β* ≈ 3, is expected to show a right-shift in approximate proportion to √t_obs_[11, 14].

(b) For real red deer data, scale-free space use (as it was defined in Figure 1A) may emerge both under the Markovian and the non-Markovian framework, and a supplementary aspect of movement has to be validated. By observing how incidence *I* (number of grid cells containing at least one fix) varied as a function of sample size N at a given spatial resolution, and then fine-tuning grid resolution to achieve normalization towards *c* = 1 in the MRW-based model *I*(N) = *c*N^*z*^, compliance with scale-free space use and the strength of site fidelity could be quantified [23]. In particular, LW and BM are expected to show *z* ≈ 1 (no return events are hindering *I* to expand proportionally with N). In other words, under the Markov framework incidence (*I*) is expected to increase approximately proportionally with N regardless of whether the movement is scale-free (LW) or not (BM). If 0 <<*z* <<1 is found (typically, in the range 0.4 < *z* < 0.6), the non-Markovian framework (as it was illustrated in Figure 1B; see Discussion) is verified [23]. In this manner, *z* is reflecting the memory aspect of movement, as defined by the *I*(N) function. As shown here, the parameter *c* in the *I*(N) function – reflecting a spatially explicit expression of site fidelity – may be estimated from the adjustment of grid resolution (the normalization procedure), as illustrated in Figure 1C.

As is shown by the present simulation results, the magnitude of *c* is not just a trivial function of grid resolution relative to the unit step length at unit time increment [expressing local responsiveness to environmental conditions as difference in movement speed, as explored in previous simulation work; e.g., Gautestad and Mysterud [25]]. Crucially for the application of MRW as model for studies on optimal search, *c* also expresses the frequency of return events relative to scale-free opportunistic moves. In other words, *c* reflects strength of site fidelity, when the effect from grid resolution, unit step length and unit time increment for the function *I*(N) are all accounted for. In this manner, the spatially explicit site fidelity parameter *c* represents the spatial ratio complement to the temporal site fidelity ratio *ρ*. See Additional file 1 for additional details on methods.

## Electronic supplementary material

Additional file 1: **Additional details on model simulations and red deer movement** http://www.movementecologyjournal.com/imedia/9839694510401541/supp1.pdf **.** (PDF 1 MB)
