# Supplementary material for: The Lévy flight foraging hypothesis: forgetting about memory may lead to false verification of Brownian motion
Source: Mov Ecol. 2013 Oct 14;1(1):9. doi: 10.1186/2051-3933-1-9 (PMC4337803; doi:10.1186/2051-3933-1-9)
Supplement: Supplementary file 1 — Additional file 1: Additional details on model simulations and red deer movement http://www.movementecologyjournal.com/imedia/9839694510401541/supp1.pdf . (PDF 1 MB) [file 40462_2013_9_MOESM1_ESM.pdf]

The Lévy flight foraging hypothesis: forgetting about memory may lead to false verification of Brownian motion

Supplementary material: Additional details on model simulations and red deer movement

Contents

Appendix 1: The MRW model, simulation methods and conditions 2

Markovian and non-Markovian memory implementation 2

Simulation of MRW 5

Appendix 2: Supplementary simulation results 9

Simulating BM 9

MRW with extreme ratio t_{ret}/t_{obs}=100:1 10

Appendix 3: GPS data of red deer... 11

Study area 11

Red deer data 11

Statistical analyses of red deer data... 12

Supplementary red deer results I: effect on β from changing t_{obs} 13

Supplementary red deer results II: fine-tuning grid resolution towards c≈1........................ 14

References (all Appendices).. 17

Appendix 1: The MRW model, simulation methods and conditions

Markovian and non-Markovian memory implementation

Though many ecologists would argue for some kind of path analysis when analyzing GPS-data in detail, it is important to realize that GPS-data are typically collected with 1 hour intervals or so, i.e., they are not exactly tracking every mechanistic step along the movement path of an animal. Hence, a statistical-mechanical model framework is a feasible approach, whether the movement is under influence of long term memory or not.

Basically, two movement classes are available to simulate memory-influenced space use; Markovian-compliant and non-Markovian. In the Markovian case a stochastic element, frequently represented by correlated random walk, is combined with deterministic rules for memory-based directed returns towards previously visited locations [1]. This implies a mixture of intrinsic directional persistence of two kinds; a tendency to keep the direction of the foregoing step (correlated random walk) and a bias towards remembered locations. At the mechanistic level the strength of the bias depends on a moment-to-moment perception of the potential foraging gain from re-visiting a specific patch versus just “move along” in a correlated random walk manner. In this model, memory is implemented in a Markovian-style manner, since both directional persistence and memory-based bias are re-calculated moment-by-moment by the actual movement algorithm. The calculation is based (a) on local conditions at the model animal’s present location, and (b) on perceived gain from moving in the direction of the assumed most profitable patch at the present point in time. Hence, this model implements memory of past patch visits by expanding the individual’s field of perception relative to its current location [2]. While the model is executed at a mechanistic scale, one could get a statistical-mechanical representation of the output by sub-sampling every n’th relocation (where n is sufficiently large). Properties of this coarser representation

of space use (which would resemble GPS fixes; see above) could then be calculated to infer indirectly about the animal's behavior.

On the other hand, to implement memory in a non-Markovian manner one could postulate that an animal might have several goals running in parallel at different temporal resolutions. In this case, a Markovian structure will not suffice, owing to the intrinsic tension between execution of high-frequency short term goals under weaker or stronger constraint of medium- and longer term goals. It is in this case not straightforward to define a specific Markovian calculation for the next step's direction and movement speed, based on moment-to-moment local conditions only (whether an element of memory-based bias towards a remembered location is superimposed on local conditions or not). Specifically, since a Markovian-structured memory model [1] includes the directional bias (the memory-dependent component) in the mechanistic step-by-step calculation of the next move, it is not possible for the model individual to execute a tactical left-directed step while the bias term of the equation is stronger than the other terms and thus determining a right-directed move towards a more distant goal. Since short term tactics and longer term strategy are superimposed in the movement algorithm that determines the next step's direction, conflicts between tactics and strategy is solved by the present "weights" of the respective terms in the movement function. These weights are re-defined in a step-wise manner; i.e., Markovian, in accordance to the conditions at the given point in time and locality in space.

To avoid this issue where tactical and strategic considerations have to lead to a specific "averaged", or "democratic" goal (all aspects considered at the present point in time and space), one may choose to shift the focus on the system by temporal coarse-graining in the first place, rather than executing the model at the mechanistic scale and then sub-sample relocations from the simulation output (as described above for the Markovian model). This approach is – basically – the outline for the Multi-scaled random walk (MRW) approach. In

this case one assumes that the simulated path is collected at a temporal resolution *t* that is large enough to ensure a statistical-mechanical level of system dynamics. In other words, *t* is assumed to be sufficiently large to embed mechanistic behavior at finer scales <<*t*. At the statistical-mechanical level, one may then postulate an alternative to the Markov-mechanistic moment-to-moment (sequential) calculation where tactics and strategic terms are simultaneously weighted against each other to produce a given result for the next move.

The MRW model postulates that tactics and strategy may run with separate goals at different temporal scales. In short, a short-term move to the right may be executed even if the longer term goal is to move to the right, given that the magnitude of such finer-scaled violations of the longer term strategy is not compromising the longer term goal. In this manner coarser-scale strategic dynamics represents a constraint on finer-scale tactics [3]. Since a movement algorithm involving both tactical and strategic terms by necessity has to be mechanistic and thus Markov-structured, the MRW framework alternatively describes movement at the coarser statistical-mechanical level to avoid the mechanistic “Markov-trap”. At this coarser level, movement tactics and strategy is expressed by a *postulate* about scale-free space use under influence of site fidelity in statistical terms, and a statistical-mechanical path algorithm that is based on this postulate. Hence, a given step calculation involves two stochastic terms; one for scale-free exploratory steps (mimicked by a LW function) and one for occasional return events to a previous location (the site fidelity aspect). The crucial point is that the MRW postulate leads to a set of predictions about space use patterns, which can be tested on real GPS data using non-scaling and/or non-memory models as null hypotheses.

A statistical-mechanical level implies an alternative set of observable quantities, which deviates qualitatively from what is observed at the mechanistic level [4]. As shown in the present results, these observables may be applied to test for memory influence on movement. It is also possible to distinguish Markovian (e.g., ref. [1]) from non-Markovian memory

execution (MRW), for example from differences in the expected fractal dimension of space use [2, 3]. However, Markovian and non-Markovian approaches to implement memory share the basic principle that movement is considered a mixture of exploratory steps and affinity towards previously visited patches.

Simulation of MRW

MRW of length 10^7 steps (8 replicates of each condition) was simulated in a homogeneous environment as a set of successively independent step vectors with length $L_{MRW}=\alpha(\mathrm{RND})^{-1/(\beta-1)}$ with $\alpha=1$ and $\beta=2$. RND is a random number between 0 and 1. Using a constant α implies a constant average step length for the simulations; i.e., movement speed (step length per unit time increment) is assumed constant on average. Inter-step direction was drawn uniformly from 0-2π radians, which implies simulation execution at a statistical mechanical meso-scale relative to the micro-scale where the actual behavioral algorithm is expressed (reflected by the fine-grained path of the animal). On average at every 10,000th, 1,000th, 100th or 10th time increment, representing respective t_{ret}, the step was replaced by a directed return to a randomly chosen previous location in the series (representing infinite memory). These variants of boundary conditions describe a progression towards smaller ranging area for a given series length, along an environment gradient with assumed increasing resource abundance. The random choice of target reflects the implicit assumption that even in an objectively homogeneous environment there may be a subjective positive fitness value connected to site fidelity (auto-facilitation from patch familiarity [5]).

The infinite memory condition above was replicated by series where memory horizon for collection of historic locations was constrained to a trailing window of last 10,000 time increments and t_{ret} varying as for the infinite memory conditions.

Step length truncation was for all conditions above set to the maximum length within the largest arena for the simulations (LW-like condition), in practice implying no truncation from other factors than return steps. Thus, series were also produced with infinite memory but physical constraint on step lengths; i.e., mimicking a truncated power law. This “physical” truncation adds to the return step effect on observed step lengths when a series is sampled at scale t_{obs} = 1,000 unit time increments. Truncation was effective in a range around L_{trunc}=200,000 length units, by discarding long steps from the following rule: A replicate length L''_x for each successive step of length L'_x (x=1, 2, 3, … , 10^4), was calculated from the formula $L''_x = (L'_x /2) + RND*(L'_x)$. If $L''_x > L_{trunc}$, the actual step was discarded and replaced by a new step, and the test was repeated. Site fidelity was varied from variations of t_{ret} as above, with return event every 10,000th, 1,000th, 100th or 10th time increment. The truncation condition reflects the situation where limitations from movement speed or environmental borders may terminate the largest displacements prematurely. If these extrinsic constraints are stronger (appearing at higher frequency) than the return step events, they will contribute additionally to limit the range of scale-free (power law) distribution of steps.

Additionally, one set of series was produced with return rate $t_{ret}=10^4$, but the target was chosen among the last 10 time increments to represent MRW mimicking memory-less and free-dispersing LW. This variant represents an interface towards classical LW. To illustrate additional transition towards a LW-like pattern when t_{ret} is increased further, one series was run with t_{ret}=100,000 and t_{obs}=1,000.

Arithmetic binning (bin size L, 2L, 3L, …) was chosen for the 10-step time horizon, for better display of the “hockey stick” pattern in the extreme tail (see results). For other distributions, log-binning was applied[6].

The narrow memory horizon route towards LW may be interpreted as an adaption to an unpredictable environment (old information becomes swiftly outdated and is ignored).

One additional simulation was also run with memory-less correlated random walk (persistence 0.5 on a scale from 0 to 1) rather than MRW. The chosen temporal lag $t_{obs}=10^3$ also for this series produces BM; i.e., directional persistence is vanishing at coarser observational scales [7].

Under all conditions the relocations (sample of fixes) were collected at $t_{obs}=10^3$, leading to $N=10^5$ collected steps pr series, and a ratio for the MRW conditions of $\rho=t_{ret}:t_{obs}$ of 10:1, 1:1, 1:10 and 1:100 respectively. Each series except for the correlated random walk case was initiated by a memory-less LW of length 10,000 steps (1,000 steps for condition $t_{ret}=10$) to represent an individual entering an unfamiliar area and then switching to memory map utilization. The first 1,000 collected steps from each series were discarded to minimize transient effects (i.e., N=9,000 for analysis).

Step length distributions were produced using standard histogram method (arithmetic binning) and power-law optimized method (geometric binning[6]). Slopes were estimated from least square regression after log transformation of both axes. Unit bin width for all series was set to 2600 length units, which represents ca 50% larger scale than median step length size for the data set with the least influence from memory. Eight replica of each of the 6 conditions were included in the analyses.

To verify memory-induced site fidelity and sub-diffusive property of MRW, the *I*(N) method, a virtual grid was superimposed onto each spatial scatter of fixes [see details under Statistical methods in connection with analysis of red deer data (below)]. Number of non-empty grid sells (embedding at least one fix) as a function of sample size N'<=N was then analyzed by regression, using the model[3] $I(N)\approx cN^z=cN^{1-D/2}$ where D is the fractal dimension. Grid resolution was adjusted to approximate $c\approx1$ for the respective boundary conditions in order to minimize statistical artifact from too large or too small grid cells (the space fill effect and the dilution effect, respectively) [8]. To study the effect of variable degree

of auto-correlation under the 6 conditions, N was varied from two methods: continuous sample size expansion (N proportional with time) and uniform expansion (N proportional with frequency within total time for the series).

Appendix 2: Supplementary simulation results

Simulating BM

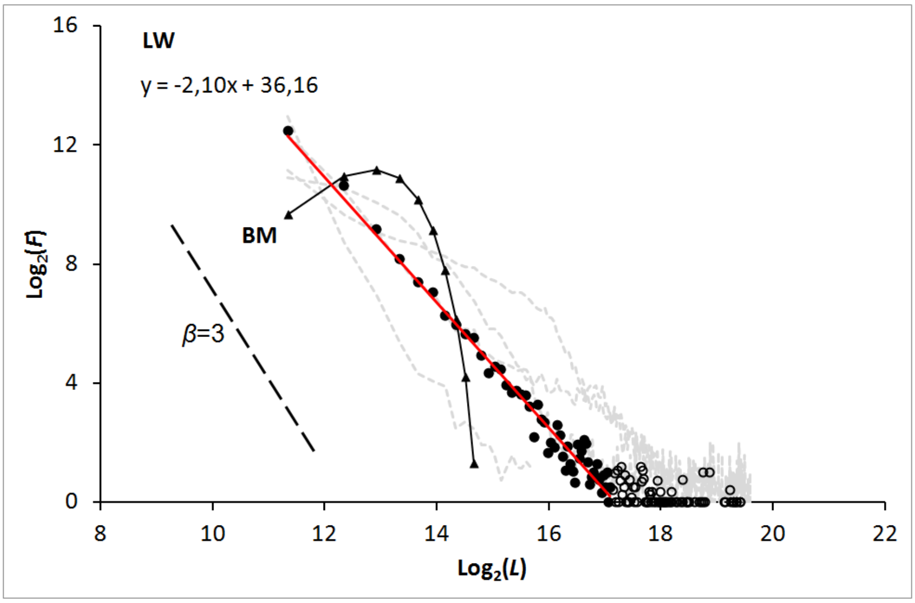

Figure A1 shows the log-transformed distribution of step lengths from the correlated random walk simulation, which produces a BM-compliant pattern when sampled at coarser scale t_{obs}*=1:10,000. Data from Figure 2 in the main text are maintained for comparison. The BM results deviates from the LW-like result by a narrower step length distribution, with no power law compliance (no zone of L with stable* β*, and* β*>>3 in the tail part).*

MRW with extreme ratio t_{ret}/t_{obs}=100:1

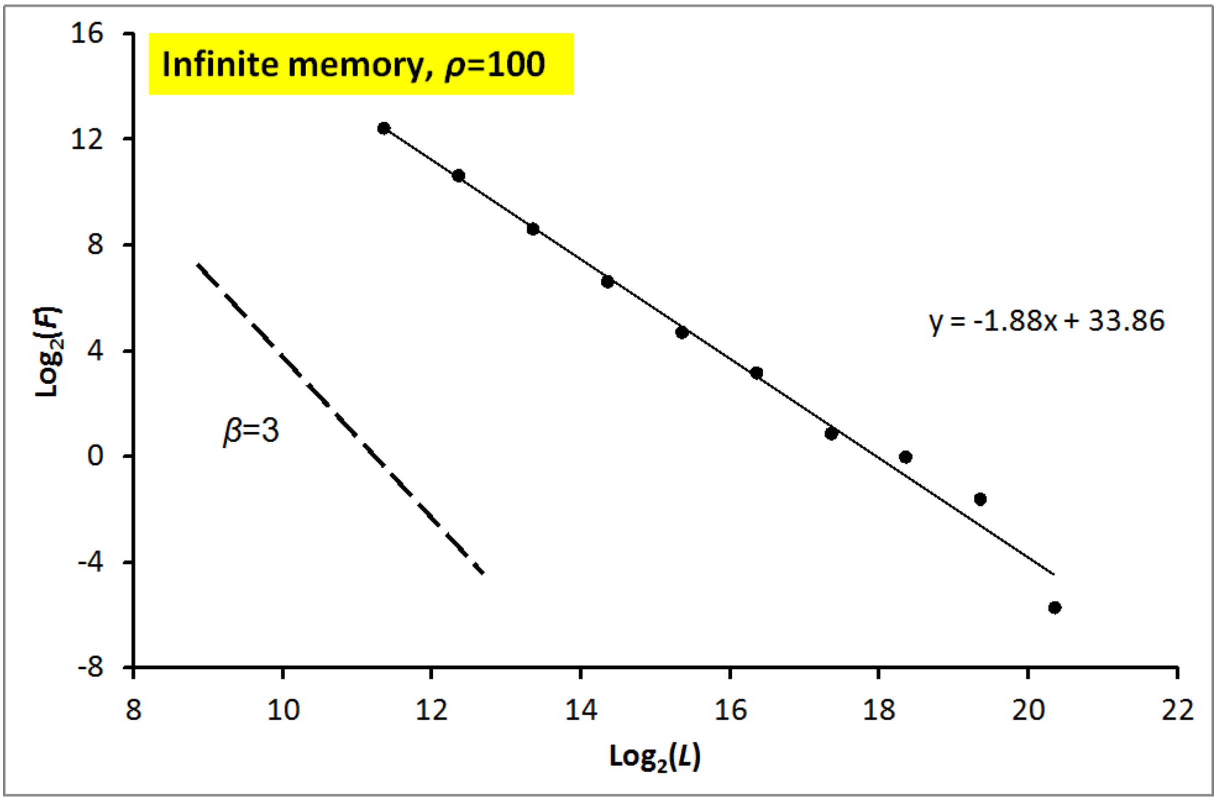

Figure A2 shows the log-transformed distribution of 9,000 step lengths (log-binning) from a simulation of MRW with t_{ret}=1:100,000 and t_{obs}=1:1,000 (implying a ratio $\rho = t_{ret}/t_{obs}$ =100:1). At this magnitude of t_{ret}/t_{obs}, the influence from return steps is close to negligible even towards large bins – and the pattern appears LW-like over the entire range of L. Truncation was set to the neighborhood of 800,000 ≈ 2^{20} length units. The slope -1.88 implies β=1.88, which is below the limit for transition towards stationary variance (the inset line for β=3 shows this transition slope, for visual comparison).

Appendix 3: GPS data of red deer

Study area

The study area is in Sogn og Fjordane county at the western part of southern Norway. The vegetation is mostly in the boreonemoral zone (Abrahamsen *et al.* 1977). Forests are dominated by deciduous (mainly birch *Betula* sp. and alder *Alnus incana*) and pine forest (*Pinus sylvestris*). Norway spruce (*Picea abies*) has been planted in many areas[9]. The terrain is characterized by valleys and mountains from coastal to inland areas. Red deer is the most common cervid in the area. In addition livestock, in particular sheep, is common in some areas. More detail description of the habitat can be found elsewhere [9].

Red deer data

The GPS data comes from 18 female red deer caught by darting at winter feeding sites during 2005-2008 (individual ID codes: SF_11, SF_14, SF_17, SF_22, SF_24, SF_25, SF_30, SF_32, SF_35, SF_37, SF_38, SF_39, SF_47, SF_49, SF_52, SF_85, SF_90 and SF_2045). All series consist of at least 2000 fixes each, collected with time lag 1 hour. The fix interval was larger due to missing positions exceeded 4 hours in only 0.6% of the observations. These 18 individuals have been verified to show power law distribution of step lengths in combination with site fidelity [2]. Hence, space use was shown to be MRW-like rather than LW-like. Here we extend the analysis by applying the parallel shift method under the Lagrangian approach (step length distribution) and adjusting grid resolution for each individual to estimate the "spatial grain unit" parameter c under the Eulerian approach (incidence as a function of sample size of fixes).

The procedure for capture was approved by the Norwegian national ethical board for science ("Forsøksdyrutvalget", http://www.fdu.no). The deer were fitted with Televilt Basic

"store-on-board" GPS (Global Positioning system) collars or Televilt Basic GPS collars with GSM (Global System for Mobile communications) option for transfer of data via cell phone network (Televilt TVP Positioning AB, Lindesberg, Sweden)[10]. The GPS collars were programmed to record hourly positions, and to release a drop-off mechanism after approximately 10 months (tracking period ranged from 6-12 months). The median location error for our GPS collars was 12 m [upper 95% CI=23 m [10]]. We removed points located further than 10 km/h from the preceding location (n=85; 0.024 % of all points), because they most likely represent large GPS errors and not true deer locations. We here use data from the summer period from June 1st – September 16th, except for four individuals where fix sampling was terminated earlier owing to abandoning of summer range and start of autumn migration [11]. Biologically this means the period when individuals are in their summer range and when most adult females give birth to a calf. Calving status of the marked red deer females was unknown, but most adult females ovulate and are likely accompanied by a calf [12]. The end of the period coincides with the onset of hunting season (from 10th September), fall migration [11], and rutting activities [13].

Statistical analyses of red deer data

To estimate super-diffusion and power law compliance from the Parallel shift method, each of the 18 series of fixes was re-sampled 1:10 (collecting every 10th relocation; t_{obs}=10 h), resulting in series lengths of ca 200-280 fixes pr individual. The distribution of step lengths F(*L*) for relocations of the pooled set of step lengths at this coarser t_{obs} (averaged over 18 series) were then compared with the average distribution from 200-280 step lengths, uniformly sampled from each of the original series at $t_{obs}\approx 1$ h (the parallel shift method requires equal sample sizes due to non-stationary distributions under condition of LW or MRW; otherwise a correction term needs to be included). Bin width was set to 95 m,

representing 50% wider bin than the median step length for the pooled series of fixes. F(*L*) was also studied for each individual, both at t_{obs}=1 h and t_{obs}=10 h. Bin width was in this case calculated specifically for each individual, and set to 50% larger than the respective median step length.

Expectation for parallel shift is $t_{2obs}^{1/(\beta-1)} / t_{1obs}^{1/(\beta-1)}$, where t_{2obs} and t_{1obs} are observational lags at coarse and fine scales, respectively [14].

Supplementary red deer results I: effect on β from changing t_{obs}

Table A1. Estimated β from lag 1 h and from 10 h, based on linear regression of log-log transformed F(*L*) histograms. Bin width was calculated specifically for each individual, and set to 50% larger than the respective median step length. For all individuals a larger lag resulted in a steeper slope (larger β), with respective mean values 1.91 and 2.45 (paired t-test, 2-sided: t=-6.39, p<0.001, 16 d.f.). No value could be obtained for SF_2045s at lag 10 h, owing to too small number of F(L) plots.

	**Lag 1 h**	**Lag 10 h**
SF_11s	1.786	1.869
SF_14s	1.851	2.579
SF_17s	1.583	1.797
SF_22s	2.372	2.778
SF_24s	1.552	2.141
SF_25s	1.726	2.545
SF_30s	2.029	2.334
SF_32s	1.898	1.905
SF_35s	1.505	1.86
SF_37s	1.862	2.185
SF_38s	2.294	3.006
SF_39s	1.909	3.012
SF_47s	2.122	2.377
SF_49s	2.155	3.245
SF_52s	2.044	2.783
SF_85s	1.917	2.303
SF_90s	1.822	2.892
SF_2045s	2.002	-

Supplementary red deer results II: fine-tuning grid resolution towards c≈1

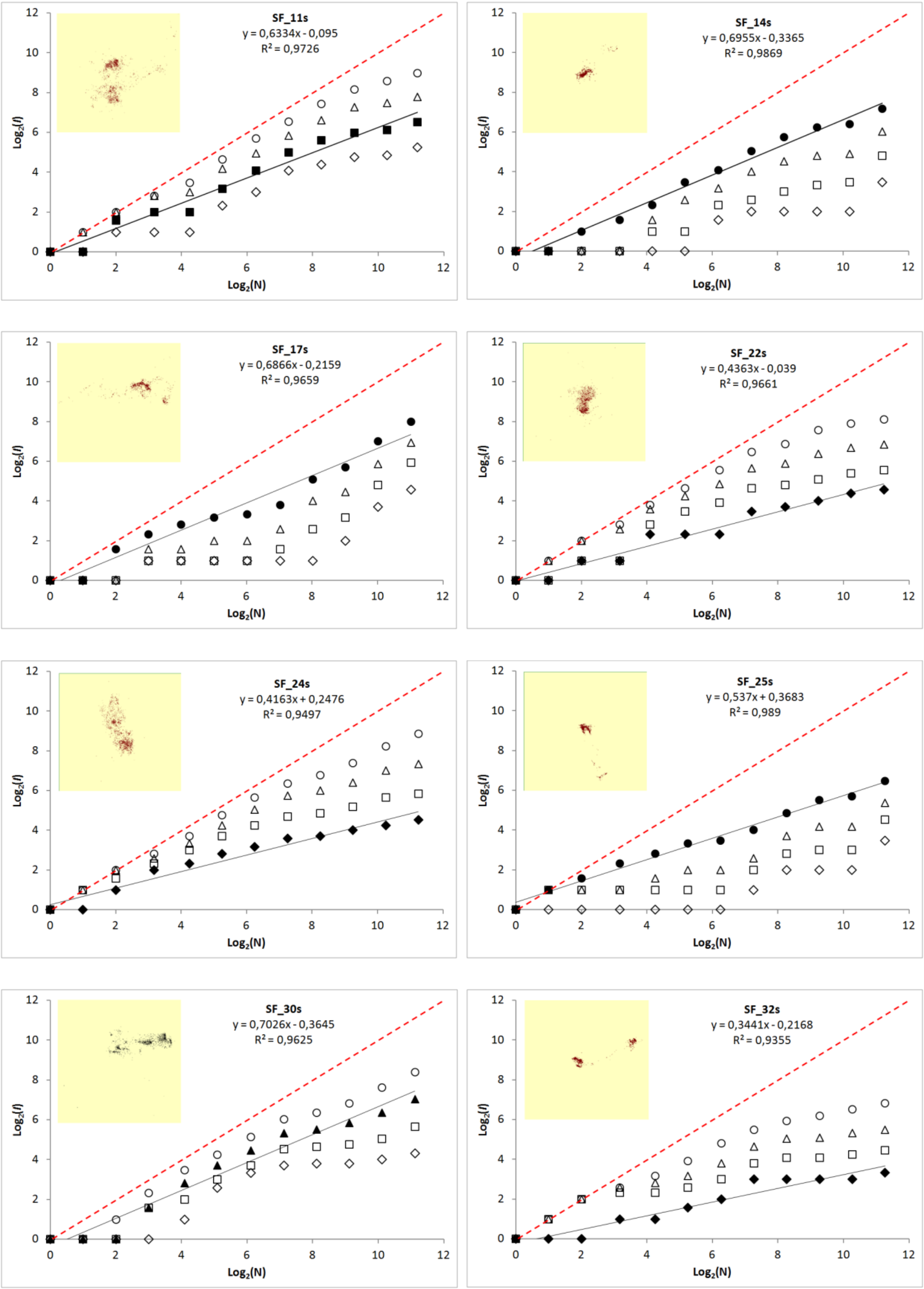

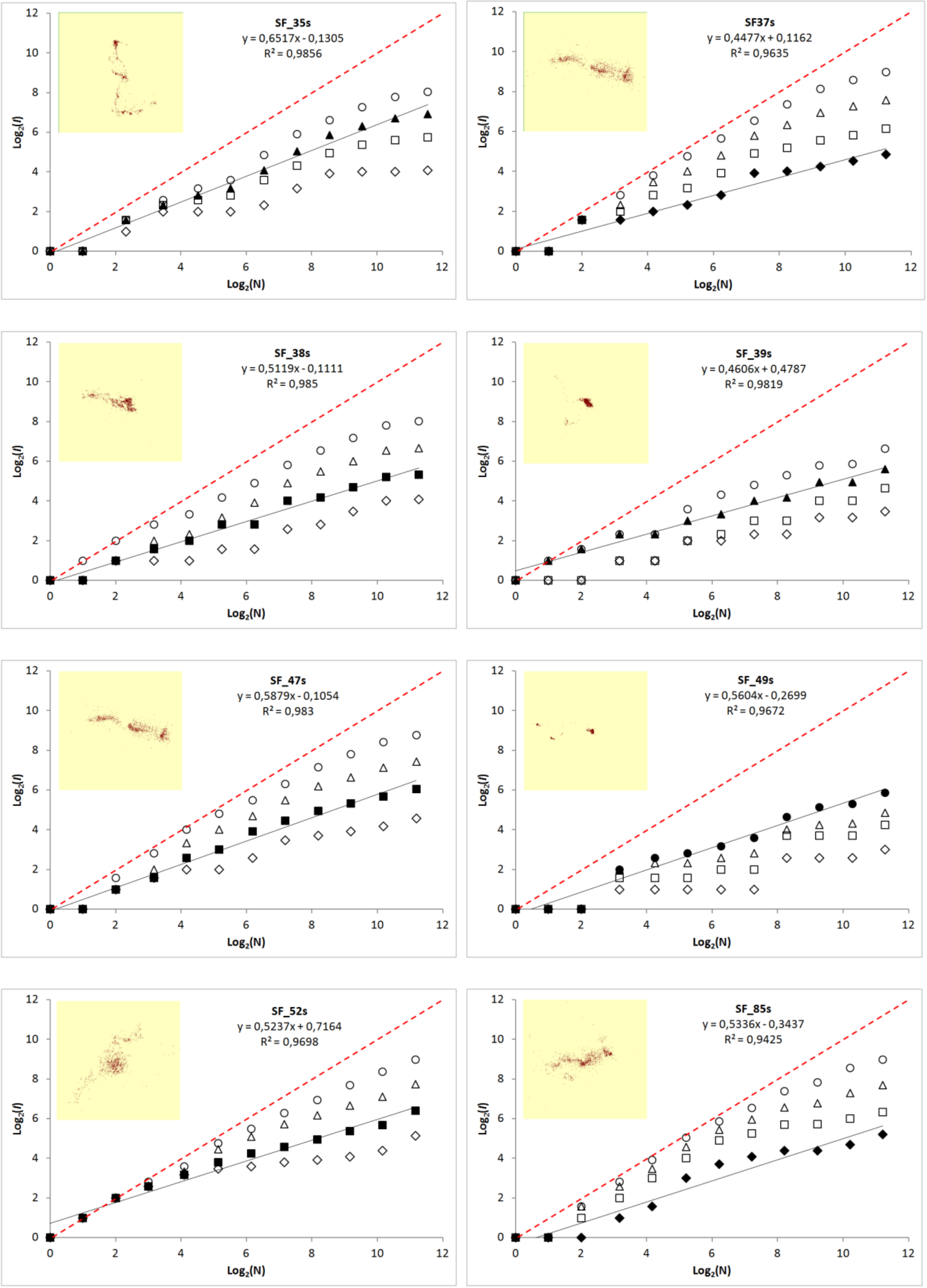
SF_35s
y = 0,6517x - 0,1305
R² = 0,9856
SF37s
y = 0,4477x + 0,1162
R² = 0,9635
SF_38s
y = 0,5119x - 0,1111
R² = 0,985
SF_39s
y = 0,4606x + 0,4787
R² = 0,9819
SF_47s
y = 0,5879x - 0,1054
R² = 0,983
SF_49s
y = 0,5604x - 0,2699
R² = 0,9672
SF_52s
y = 0,5237x + 0,7164
R² = 0,9698
SF_85s
y = 0,5336x - 0,3437
R² = 0,9425
Log₂(I)
Log₂(N)

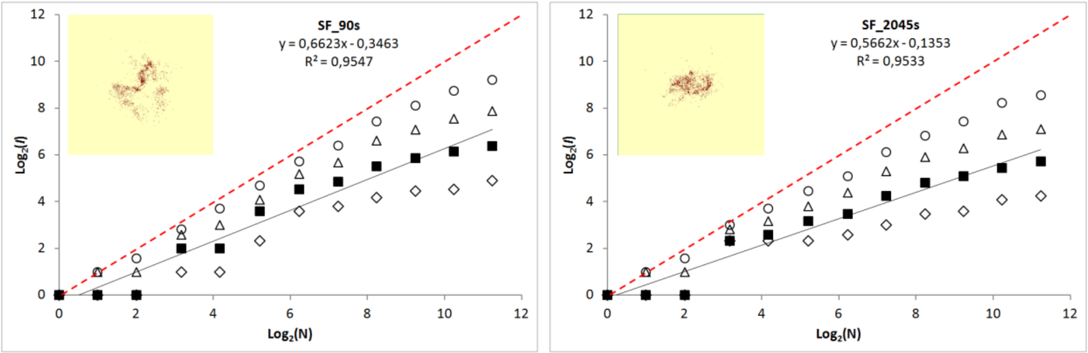

Figure A3. The 18 series of red deer fixes were subject to I(N) analysis, where $I(N) \approx cN^z$ (see main text). Sample size, N, was increased successively in a geometric manner (1, 2, 4, 8, ..., N_{max}). Grid cells containing at least one fix (incidence, I) was counted for each magnitude of N. Grid resolution (pixel size) R was varied to achieve c≈1 [log(c)≈0] relative to the chosen arena size embedding the respective sets of fixes. In the respective sub-figures (log-transformed axes), R=1:80, 1:40, 1:20 and 1:10 (circles, triangles, squares and diamonds, respectively). The best-fitting resolution, representing an approximation of c=1, is indicated by filled symbols. If in doubt between two pixel sizes, we chose the resolution that showed the largest R^2 statistic of the two under linear regression. Under this normalization procedure, the respective estimates of z should reflect whether the individual in question showed site fidelity (z<<1). Under this condition, the individual estimate of c (in spatial scale unit like m^2) reflects the respective (over-all) characteristic unit space use scale under the given habitat conditions and over the given sampling period. In general, a larger c (estimated under condition z<<1) reflects a more extensive space use for a given z, N and average movement speed.

References (all Appendices)

1. Van Moorter B, Visscher D, Benhamou S, Börger L, Boyce MS, Gaillard J-M: **Memory keeps you at home: a mechanistic model for home range emergence.** *Oikos* 2009, **118:**641-652.

2. Gautestad AO, Loe LE, Mysterud A: **Inferring spatial memory and spatiotemporal scaling from GPS data: comparing red deer Cervus elaphus movements with simulation models.** *Journal of Animal Ecology* 2013, **82:**572-586.

3. Gautestad AO, Mysterud I: **The home range fractal: from random walk to memory dependent space use.** *Ecological Complexity* 2010, **7:**458-470.

4. Gautestad AO: **Memory matters: Influence from a cognitive map on animal space use.** *Journal of Theoretical Biology* 2011, **287:**26-36.

5. Gautestad AO, Mysterud I: **Spatial memory, habitat auto-facilitation and the emergence of fractal home range patterns.** *Ecological Modelling* 2010, **221:**2741-2750.

6. Sims DW, Righton D, Pitchford JW: **Minimizing errors in identifying Lévy flight behaviour of organisms.** *Journal of Animal Ecology* 2007, **76:**222-229.

7. Turchin P: *Quantitative analysis of animal movement: measuring and modeling population redistribution in animals and plants.* Sunderland, Massachusetts: Sinauer Associates; 1998.

8. Gautestad A, Mysterud I: **The Dilution Effect and the Space Fill Effect: Seeking to Offset Statistical Artifacts When Analyzing Animal Space Use from Telemetry Fixes.** *Ecological Complexity* 2012, **9:**33-42.

9. Mysterud A, Langvatn R, Yoccoz NG, Stenseth NC: **Large-scale habitat variability, delayed density effects and red deer populations in Norway.** *Journal of Animal Ecology* 2002, **71:**569-580.

10. Rivrud IM, Loe LE, Mysterud A: **How does local weather predict red deer home range size at different temporal scales?** *Journal of Animal Ecology* 2010, **79:**1280-1295.

11. Mysterud A, Loe LE, Zimmermann B, Bischof R, Veiberg V, Meisingset EL: **Partial migration in expanding red deer populations at northern latitudes – a role for density dependence?** *Oikos* 2011, **120:**1817-1825.

12. Langvatn R, Mysterud A, Stenseth NC, Yoccoz NG: **Timing and synchrony of ovulation in red deer constrained by short northern summers.** *American Naturalist* 2004, **163:**763-772.

13. Loe LE, Bonenfant C, Mysterud A, Gaillard J-M, Langvatn R, Stenseth NC, Klein F, Calenge C, Ergon T, Pettorelli N: **Climate predictability and breeding phenology in red deer: timing and synchrony of rutting and calving in Norway and France.** *Journal of Animal Ecology* 2005, **74:**579-588.

14. Gautestad AO: **Brownian motion or Lévy walk? Stepping towards an extended statistical mechanics for animal locomotion.** *Journal of the Royal Society Interface* 2012, **9:**2332-2340.
